# Brain architecture of the largest living land arthropod, the Giant Robber Crab *Birgus latro *(Crustacea, Anomura, Coenobitidae): evidence for a prominent central olfactory pathway?

**DOI:** 10.1186/1742-9994-7-25

**Published:** 2010-09-10

**Authors:** Jakob Krieger, Renate E Sandeman, David C Sandeman, Bill S Hansson, Steffen Harzsch

**Affiliations:** 1 Institute of Zoology, Department of Cytology and Evolution, University of Greifswald, Johann-Sebastian-Bach-Straße 11/12, D-17487 Greifswald, Germany; 2 Justus-Liebig-Universität Gießen, Fachbereich 06 Psychologie und Sportwissenschaft, Abteilung für Entwicklungspsychologie, Otto-Behaghel-Strasse 10F, D-35394 Giessen, Germany; 3 Wellesley College, 106 Central Street, Wellesley College, Department of Biological Sciences, Wellesley, MA 02481-8203, USA; 4 Max Planck Institute for Chemical Ecology, Department of Evolutionary Neuroethology, Beutenberg Campus, Hans-Knöll-Str. 8, D-07745 Jena, Germany

## Abstract

**Background:**

Several lineages within the Crustacea conquered land independently during evolution, thereby requiring physiological adaptations for a semi-terrestrial or even a fully terrestrial lifestyle. *Birgus latro *Linnaeus, 1767, the giant robber crab or coconut crab (Anomura, Coenobitidae), is the largest land-living arthropod and inhabits Indo-Pacific islands such as Christmas Island. *B. latro *has served as a model in numerous studies of physiological aspects related to the conquest of land by crustaceans. From an olfactory point of view, a transition from sea to land means that molecules need to be detected in gas phase instead of in water solution. Previous studies have provided physiological evidence that terrestrial hermit crabs (Coenobitidae) such as *B. latro *have a sensitive and well differentiated sense of smell. Here we analyze the brain, in particular the olfactory processing areas of *B. latro*, by morphological analysis followed by 3 D reconstruction and immunocytochemical studies of synaptic proteins and a neuropeptide.

**Results:**

The primary and secondary olfactory centers dominate the brain of *B. latro *and together account for ca. 40% of the neuropil volume in its brain. The paired olfactory neuropils are tripartite and composed of more than 1,000 columnar olfactory glomeruli, which are radially arranged around the periphery of the olfactory neuropils. The glomeruli are innervated ca. 90,000 local interneurons and ca. 160,000 projection neurons per side. The secondary olfactory centers, the paired hemiellipsoid neuropils, are targeted by the axons of these olfactory projection neurons. The projection neuron axonal branches make contact to ca. 250.000 interneurons (per side) associated with the hemiellipsoid neuropils. The hemiellipsoid body neuropil is organized into parallel neuropil lamellae, a design that is quite unusual for decapod crustaceans. The architecture of the optic neuropils and areas associated with antenna two suggest *that B. latro *has visual and mechanosensory skills that are comparable to those of marine Crustacea.

**Conclusions:**

In parallel to previous behavioral findings that *B. latro *has aerial olfaction, our results indicate that their central olfactory pathway is indeed most prominent. Similar findings from the closely related terrestrial hermit crab *Coenobita clypeatus *suggest that in Coenobitidae, olfaction is a major sensory modality processed by the brain, and that for these animals, exploring the olfactory landscape is vital for survival in their terrestrial habitat. Future studies on terrestrial members of other crustacean taxa such as Isopoda, Amphipoda, Astacida, and Brachyura will shed light on how frequently the establishment of an aerial sense of olfaction evolved in Crustacea during the transition from sea to land. Amounting to ca. 1,000,000, the numbers of interneurons that analyse the olfactory input in *B. latro *brains surpasses that in other terrestrial arthropods, as e.g. the honeybee *Apis mellifera *or the moth *Manduca sexta*, by two orders of magnitude suggesting that *B. latro *in fact is a land-living arthropod that has devoted a substantial amount of nervous tissue to the sense of smell.

## Background

**Figure 1:**
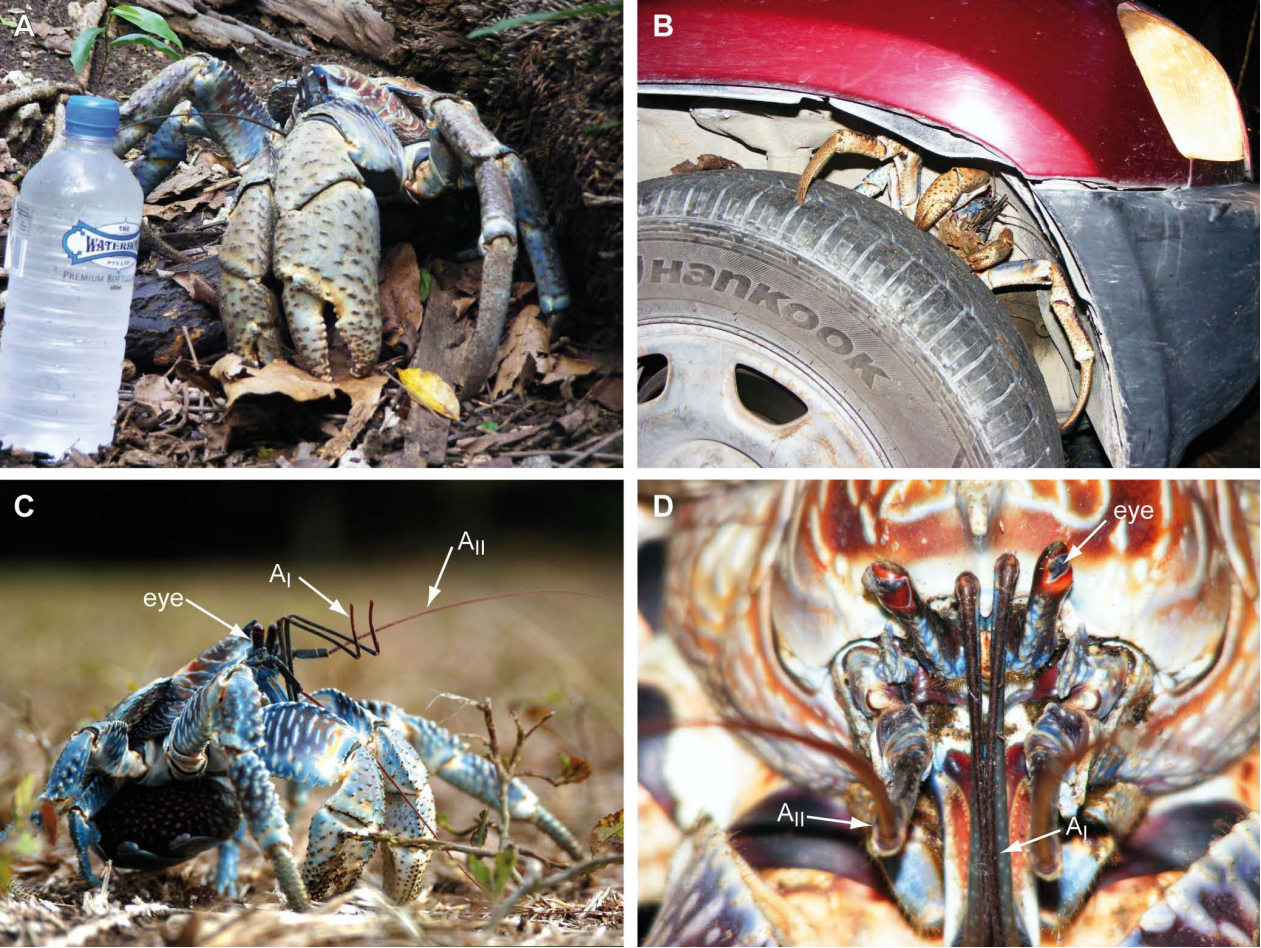
***Birgus latro *on Christmas Island**. **A-D**: Digital photographs of *Birgus latro *in the rainforest on Christmas Island. Abbreviations: AI, antenna 1 (antennules); AII, antenna 2.

Within the anomuran crustaceans, the Coenobitidae (terrestrial hermit crabs) have succeeded in the transition from an aquatic to a fully terrestrial life style and have developed pronounced terrestrial adaptations [1-3] that, apart from for the larval stages, allow them to permanently inhabit supralitoral areas and small islands of tropical and subtropical maritime regions, and to penetrate long distances inland [4,5]. These crabs are members of the Paguroidea ("hermit crabs"), a taxon, the members of which have evolved the potential to protect the pleon with gastropod shells. The Coenobitidae comprise two genera, where all species display a fully terrestrial life style [6]. These include 15 species of shell-carrying land hermit crabs (the genus *Coenobita*) and the robber or coconut crab *Birgus latro *Linnaeus, 1767 (monospecific genus *Birgus*) that only carries a shell as juvenile. *B. latro *is the largest living terrestrial arthropod (Fig. 1) with individuals recorded to weigh up to 4 kg and measure 200 mm in carapace width [5,7-10]. This animal is considered a grade T4 terrestrial species [5], still dependent on water for the marine pelagic larvae that spend three to four weeks at sea before migrating to terrestrial habitats. The early juvenile stages that emerge on land carry a shell, but with subsequent growth the thorax and pleon harden for protection as in other Crustacea [10], making the use of gastropod shells superfluous. *B. latro *is widely distributed on remote tropical islands of the Indian and Pacific Oceans [11-13]. At present its range includes islands off the east coast of Africa near Zanzibar and eastward to the Gambier Islands in the east Pacific. The borders of the tropical zone (latitudes: 23.4°N and 23.4°S) limit their extent to the north and south [10].

Land-living crustaceans are fascinating animals that have adapted to a number of diverse terrestrial habitats in which they have become highly successful, and in some case the predominant life forms. This has occurred during a very limited time period in an evolutionary perspective. The successful transition from marine to terrestrial life requires a number of physiological adaptations that are important for survival out of water (see reviews [1-3,5,14-16]). *B. latro *has served as a model in numerous studies of such physiological aspects, for example, gas exchange, salt and water balance, nitrogenous excretion, thermoregulation, molting and reproduction (Morris, Greenaway and their colleagues [17-35]). Living on land also raises new questions regarding the evolution of olfaction, as a transition from sea to land means that molecules need to be detected in gas phase instead of in water solution. Furthermore, the odor stimulus changes from mainly hydrophilic molecules in aqueous solution to mainly hydrophobic in the gaseous phase (review Hansson et al. [36]). Nevertheless, behavioral studies have provided evidence that members of the Coenobitidae are very effective in detecting food from a distance and in responding to airborne odors. In short, they have evolved a sense of distance olfaction that is behaviorally highly relevant [37-39]. Recently it was also shown that brain areas responsible for processing olfactory stimuli are enlarged in *Coenobita clypetaus *[40] in comparison to other decapods. As for the giant robber crab *B. latro*, electro-antennographic studies with the well developed first antennae demonstrated the capacity of this organ to detect volatile chemical information (review Hansson et al. [36]). As in other decapod crustaceans [41,42] the olfactory sensory neurons of *B. latro *are associated with specialized structures on the first pair of antennae, the aesthetascs [37]. Stensmyr et al. [37] demonstrated that the first antenna responded to CO_2 _and also noted a pronounced response to water vapor. They also tested various other volatile compounds such as dimethyl-disulfide and dimethyl-trisulfide (both emitted from decaying meat), ethyl-hexanoate (pineapple), isoamyl acetate (banana), phenylacetaldehyde (flower fragrance) and gamma-decalactone (coconut). In particular, the tested oligosulfide compounds were detected in minute concentrations [37]. These authors concluded that their experiments on *B. latro *point to a peripheral olfactory system as sensitive as the most sensitive general odor-detecting olfactory sensory neurons found in insects, and that therefore is well suited to explore the terrestrial olfactory landscape. Because a pioneering study on the brain of *B. latro *had already suggested the presence of dominating olfactory centers [43], the current study sets out to explore the general brain architecture in this species in more detail with special reference to the central olfactory pathway.

## Results

The data presented in this study are drawn from a series of silver-impregnated (Bodian) horizontal sections (compare Sandeman et al. [43]) that served as the basis for a 3 D reconstruction of the brain, a set of triple-labeled immunofluorescence experiments (the neuropeptide allatostatin, synaptic proteins, and nuclei) and an immunolocalization experiment of the neuropeptide SIFamide (see material and methods). A total of four brains were processed for immunohistochemistry. The following color-coded abbreviations identify the markers in the figures:

AST: Allatostatin-like immunoreactivity

NUC: nuclear counter-stain with the HOECHST nuclear dye

SIF: SIFamide-like immunoreactivity

SYN: synapsin immunoreactivity

**Figure 2:**
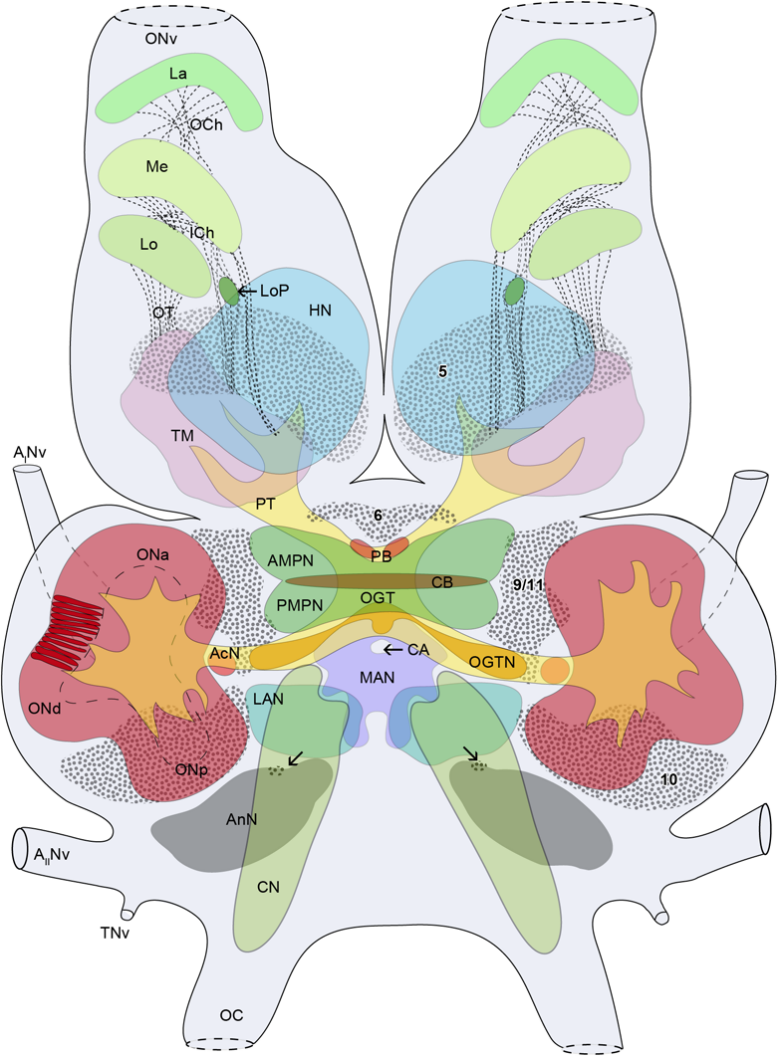
**Schematic drawing of the brain of *B. latro *(dorsal view), idealized from ca**. 40 horizontal silver-stained sections (10 μm) and several immunostained sections of (100 μm) of four animals. Abbreviations: arrows: deutocerebral organ; 5, 6, 9, 10 identify cell clusters; A_I_Nv, antenna I nerve; A_II_Nv, antenna II nerve; AcN, accessory neuropil; AMPN, anterior medial protocerebral neuropil; AnN, antenna II neuropil; CA, cerebral artery; CB, central body neuropil; CN, columnar neuropil; Me, medulla (external medulla); HN, hemiellipsoid neuropil; ICh, inner chiasma; La, lamina; LAN, lateral antenna I neuropil; Lo, lobula (internal medulla); LoP, lobula plate; MAN, median antenna I neuropil; OC, oesophageal connective; OCh, outer chiasma; OGT, olfactory globular tract; OGTN, olfactory globular tract neuropil; ONa, ONd and ONp, anterior, dorsal and posterior sublobe of the olfactory neuropil ON (shown at the left hemisphere of the brain, more detailed with columnar olfactory glomeruli); ONv, optic nerve; OT, optic tract; PB, protocerebral bridge; PMPN, posterior medial protocerebral neuropil; PT, protocerebral tract; TM, terminal medulla (medulla terminalis); TNv, tegumentary nerve.

The study provides description of the brain anatomy with a level of resolution sufficient for the establishment of a general scheme of the *B. latro *brain (Fig. 2) and for a more detailed description of the lateral and medial protocerebrum, through the deutocerebrum to the tritocerebrum. The sex was determined for all animals studied in the triple-labeling experiments, but sex-specific differences could not be detected. For simplicity, the description of the brain compartments, most of which are bilaterally paired, is limited to only one hemisphere (left side). The data resulting from the morphometric analysis are presented in combination with data by Beltz et al. ([44] see Table. 1 and 2).

**Table 1:** Morphometric analysis of olfactory compartments

Brain compartment	Neuropil	Neuropil volume (μm^3^)	Proportion of the total neuropil volume (%)	Cell cluster	Nucleus diameter (μm)	Cell counts
Entire brain		2,849,638,500	100	-	-	-

Protocerebrum	HN - hemiellipsoid body neuropil	191,421,900	13.4	5	5.55	253556
Deutocerebrum	ON - olfactory neuropil	374,681,700	26.3	9/11	6.74	89808
Deutocerebrum	AcN - accessory neuropil	500,693	0.04	10	5.43	162250

Means of olfaction-related neuropil volumes, estimated cell counts and ranges of cell nucleus diameters for corresponding cell clusters are summarized here. All values, except the percentage of the neuropil volume to the total neuropil volume of the entire brain, refer to means of a single brain hemisphere of *Birgus latro*.

**Table 2:** Morphometric comparative table

Taxon	Species	ON neuropil volume (μm^3^)	Glomerular volume (μm^3^)	Glomerular number	Aesthetasc count	Convergence ratio (aesthetasc/glomeruli)
Achelata	*Panulirus interruptus*	344,922,004	287,884	1,202	1,786	1.486
	*Panulirus argus*	154,068,687	117,862	1,332	1,255	0.942
	*Jasus edwardsii*	591,956,438	616,475	961	1,537	1.599
Homarida	*Homarus americanus*	141,159,589	591,583	249	1,262	5.068
Astacida	*Cherax destructor*	24,187,019	110,975	230	130	0.565
	*Cherax quadricarinatus*	24,735,814	74,298	334	237	0.710
	*Procambarus clarkii*	9,790,377	19,585	503	133	0.264
Thalassinida	*Callianassa australiensis*	6,588,788	28,041	235	22	0.094
Anomura	*Coenobita clypeatus*	120,352,292	153,833	799	519	0.650
	***Birgus latro***	**374,681,700**	**280,103**	**1,338**	(*780) 1700	(*0.58) 1.27
	*Petrolisthes coccnicus*	12,359,013	18,947	655	328	0.501
Brachyura	*Cancer borealis*	165,730,818	229,666	733	540	0.737
	*Libinia dubia*	20,327,317	39,338	454	319	0.703
	*Percnon planissimum*	28,765,244	58,705	495	555	1.121
	*Sesarma sp*.	6,617,077	14,887	446	33	0.074
	*Uca minax*	4,558,497	17,779	284	39	0.137
	*Uca pugilator*	3,114,604	13,14	234	28	0.120
	*Uca pugnax*	3,012,080	8,034	374	26	0.070

Means of olfactory neuropil volume, glomerular volume, calculated glomerular number, aesthetasc count and the convergence ratio per hemi-brain adopted from Beltz et al. 2003 [44] except *B. latro*. (*aesthetasc count adopted from Harms, 1932 [7]).

### Overview of the *Birgus latro *brain

**Figure 3:**
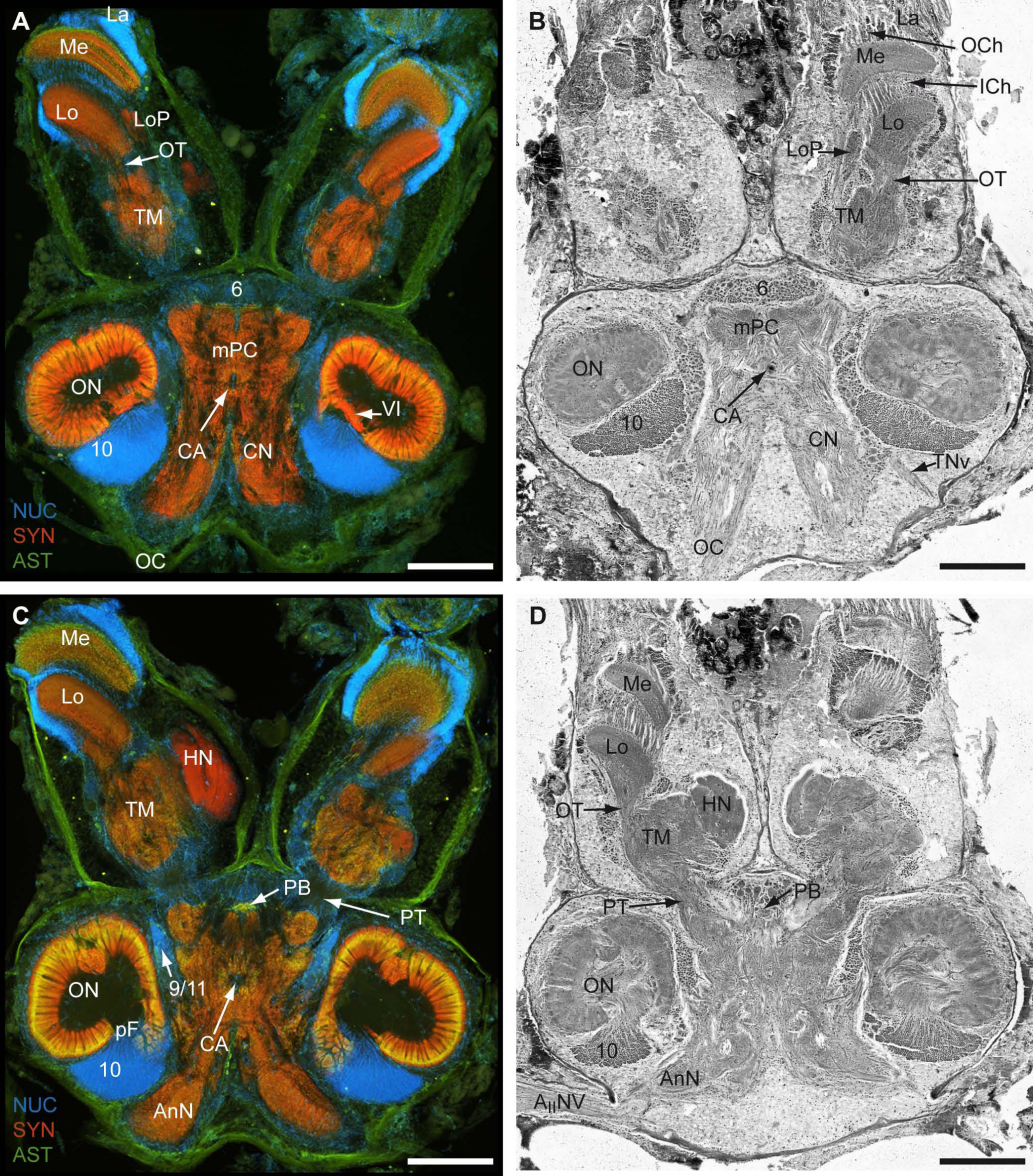
**1^st ^part of a dorsal to ventral series of vibratome sections (100 μm) triple labeled for the nuclear marker (NUC; blue), synapsin immunoreactivity (SYN; red) and allatostatin-like immunoreactivity (AST; green) (A and C) and series of silver impregnated microtome sections (10 μm) (B and D)**. **A to D**: Photomontages of 2 × 3 pictures per slice. Corresponding pictures of equivalent horizons are shown in A and B and further ventrally C and D. Roman numeral VI identifies unstructured neuropil; Numbers 6, 10 and 9/11 identify cell clusters. Other abbreviations: A_II_Nv, antenna II nerve; AnN, antenna II neuropil; CA, cerebral artery; CN, columnar neuropil, HN, hemiellipsoid neuropil; ICh, inner optic chiasm; La, lamina; Lo, lobula; LoP, lobula plate; M, medulla; mPC, medial protocerebrum; OC, oesophageal connective; OCh, outer optic chiasm; ON, olfactory neuropil (olfactory lobe); OT, optic tract; PB, protocerebral bridge; pF, posterior foramen; PT, protocerebral tract; TM, terminal medulla; TNv tegumentary nerve. Scale bar = 500 μm.

**Figure 4:**
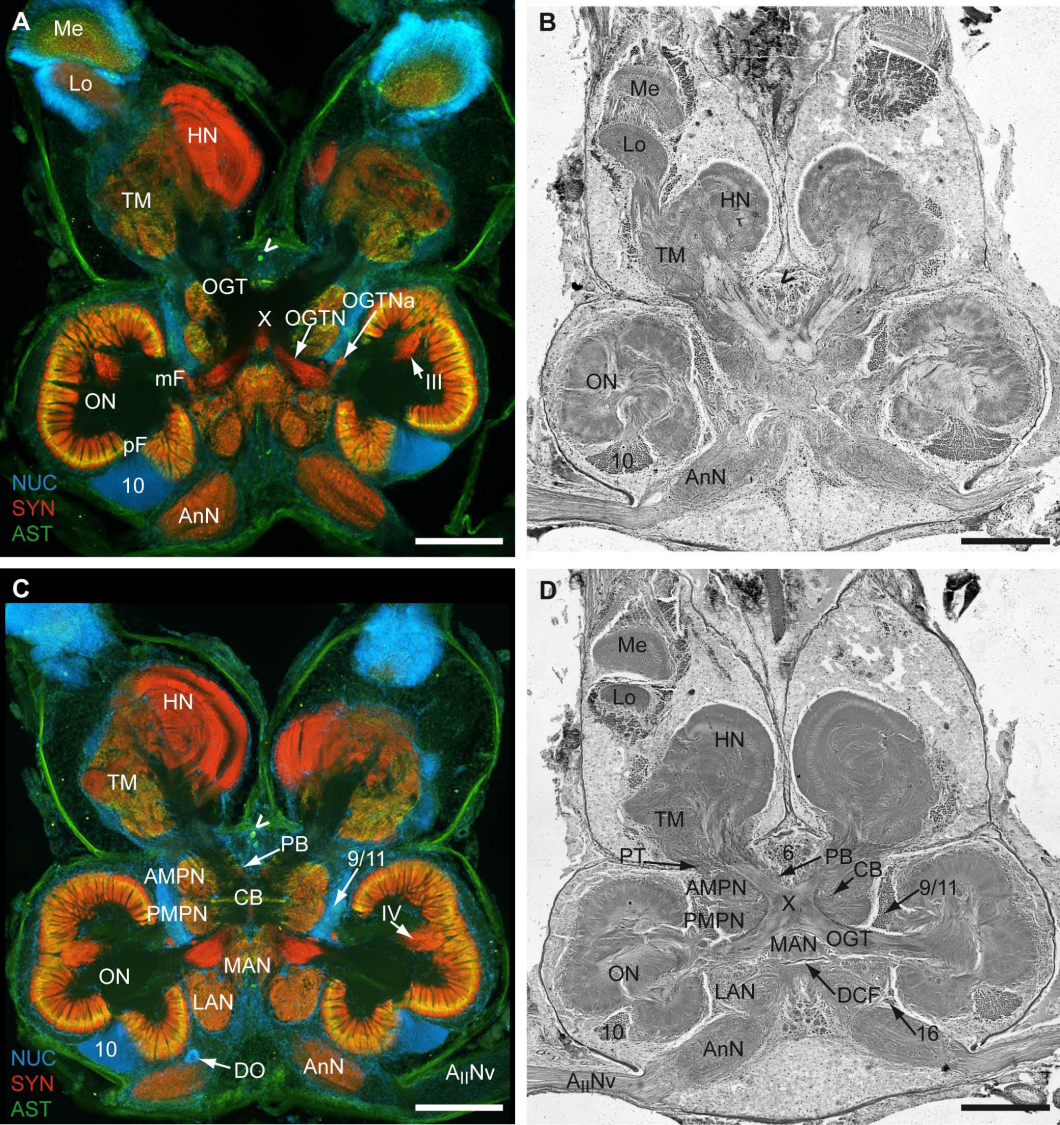
**2^nd ^part of a dorsal to ventral series of vibratome sections (100 μm) triple labeled for the nuclear marker (NUC; blue), synapsin immunoreactivity (SYN; red) and allatostatin-like immunoreactivity (AST; green) (A and C) and series of silver impregnated microtome sections (10 μm) of an equivalent horizon (B and D)**. **A to D**: Photomontages of 2 × 3 pictures per slice. Corresponding pictures of equivalent horizons are shown in A and B and further ventrally C and D. Roman numerals III and IV identify unstructured neuropils; numbers 6, 10, 9/11 and 16 identify cell clusters. Other abbreviations: Arrowheads, single ASTir neurons; A_II_Nv, antenna II nerve; AMPN, anterior medial protocerebral neuropil; AnN, antenna II neuropil; CB, central body neuropil; DCF, deutocerebral commissural fibers; HN, hemiellipsoid neuropil; LAN, lateral antenna I neuropil; Lo, lobula; M, medulla; MAN, median antenna I neuropil; mF, median foramen; DO, deutocerebral organ; OGT, olfactory globular tract; OGTN, olfactory globular tract neuropil; OGTNa, accessory olfactory globular tract neuropil; ON, olfactory neuropil (olfactory lobe); PB, protocerebral bridge; pF, posterior foramen; PMPN, posterior medial protocerebral neuropil; PT, protocerebral tract; TM, terminal medulla; X, chiasm of the OGT. Scale bar = 500 μm.

**Figure 5:**
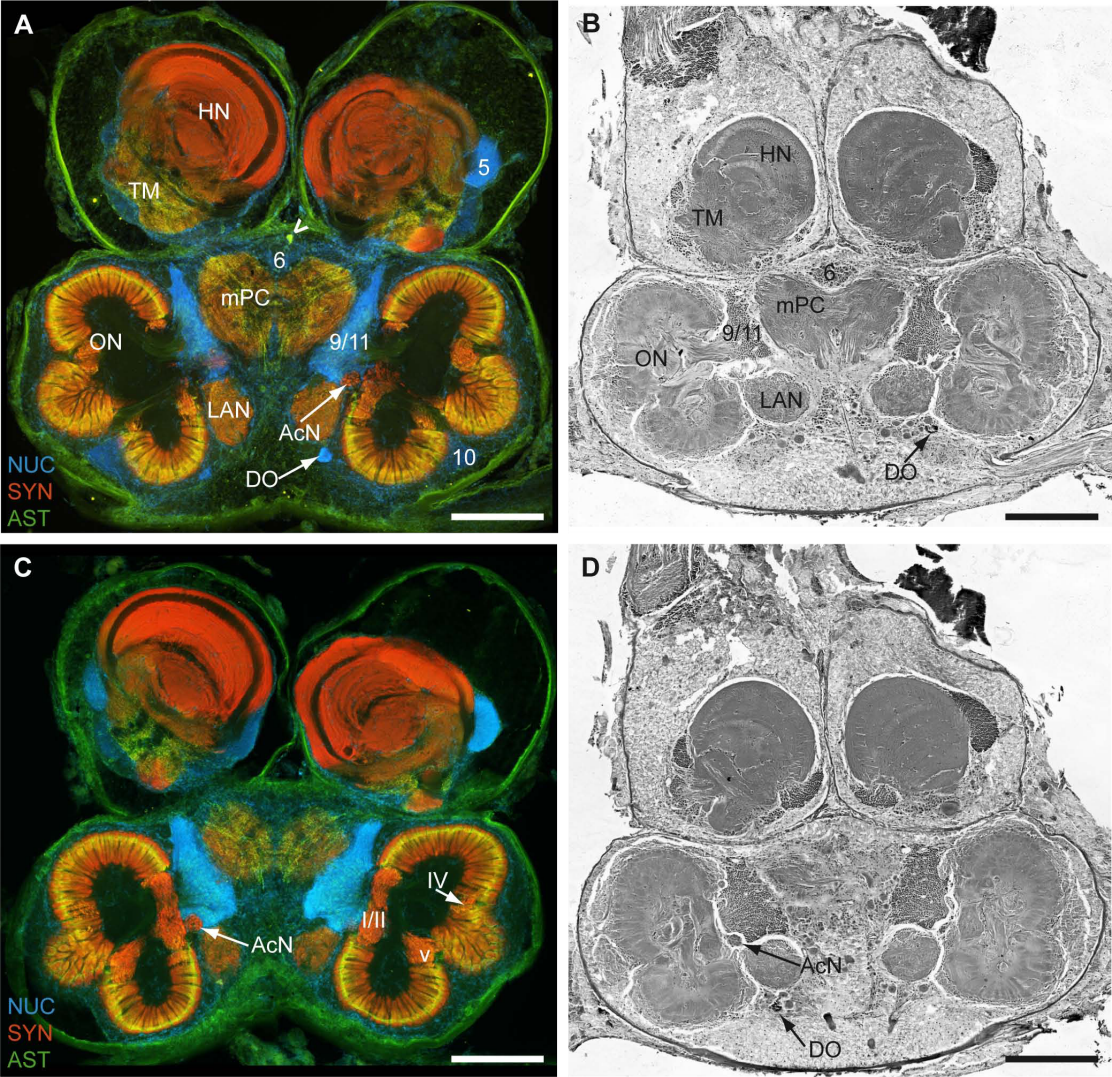
**3^rd ^part of a dorsal to ventral series of vibratome sections (100 μm) triple labeled for the nuclear counter marker (NUC; blue), synapsin immunoreactivity (SYN; red) and allatostatin-like immunoreactivity (AST; green) (A and C) and series of silver impregnated microtome sections (10 μm) of an equivalent horizon (B and D)**. **A to D**: Photomontages of 2 × 2 pictures per slice. Corresponding pictures of equivalent horizons are shown in A and B and further ventrally C and D. Roman numerals I/II, IV and V identify unstructured neuropils; numbers 5, 6, 10 and 9/11 identify cell clusters. Other abbreviations: Arrowhead, single ASTir neuron; AcN, accessory neuropil; HN, hemiellipsoid neuropil; LAN, lateral antenna I neuropil; mPC, medial protocerebrum; DO, deutocerebral organ; ON, olfactory neuropil (olfactory lobe); TM, terminal medulla. Scale bar = 500 μm.

**Figure 6:**
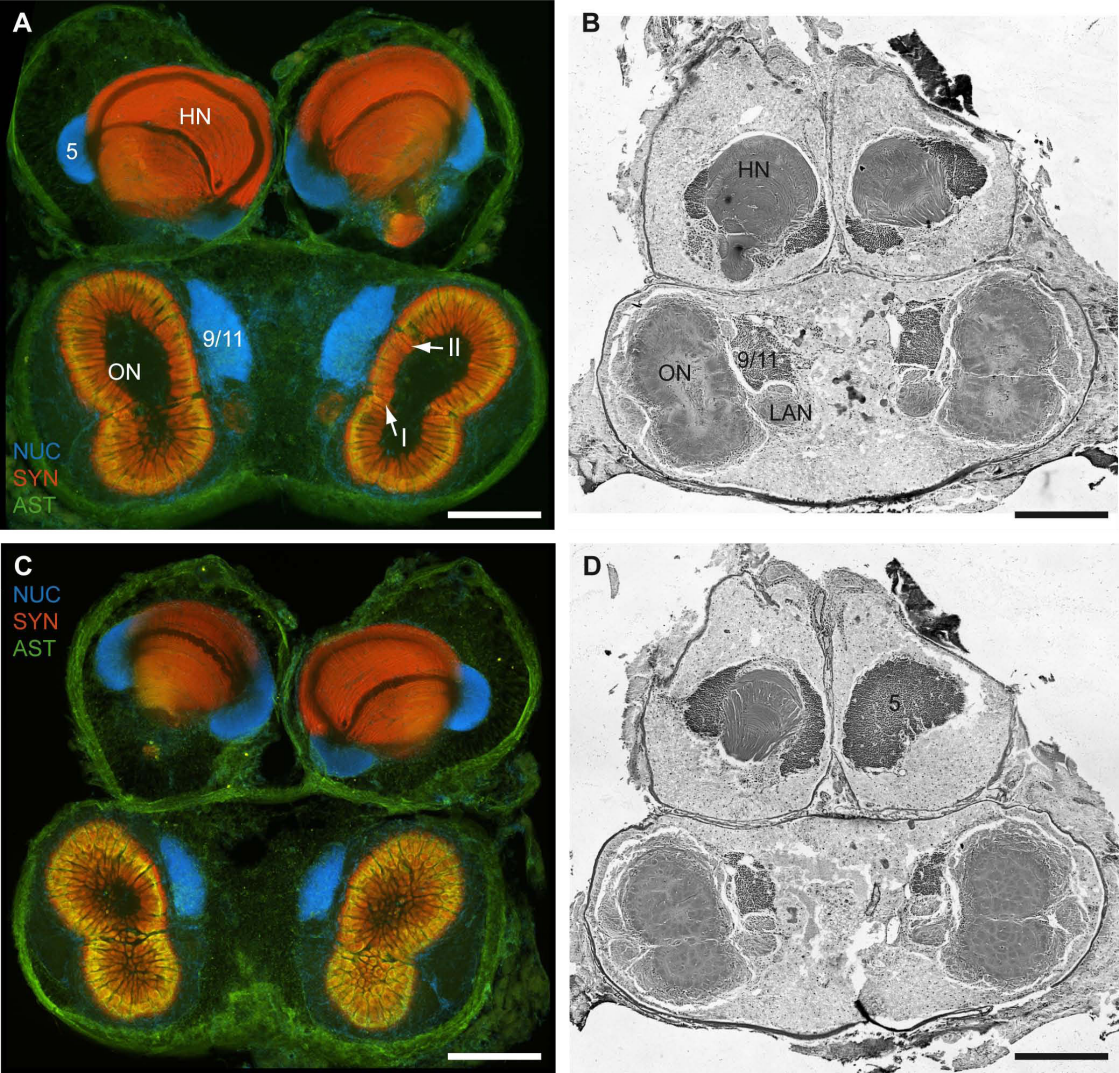
**4^th ^part of a dorsal to ventral series of vibratome sections (100 μm) triple labeled for the nuclear marker (NUC; blue), synapsin immunoreactivity (SYN; red) and allatostatin-like immunoreactivity (AST; green) (A and C) and series of silver impregnated microtome sections (10 μm) of an equivalent horizon (B and D)**. **A to D**: Photomontages of 2 × 2 pictures per slice. Corresponding pictures of equivalent horizons are shown in A and B and further ventrally C and D. Roman numerals I and II identify unstructured neuropils; numbers 5 and 9/11 identify cell clusters. Other abbreviations: HN, hemiellipsoid neuropil; LAN, lateral antenna I neuropil; ON, olfactory neuropil (olfactory lobe); Scale bar = 500 μm.

**Figure 7:**
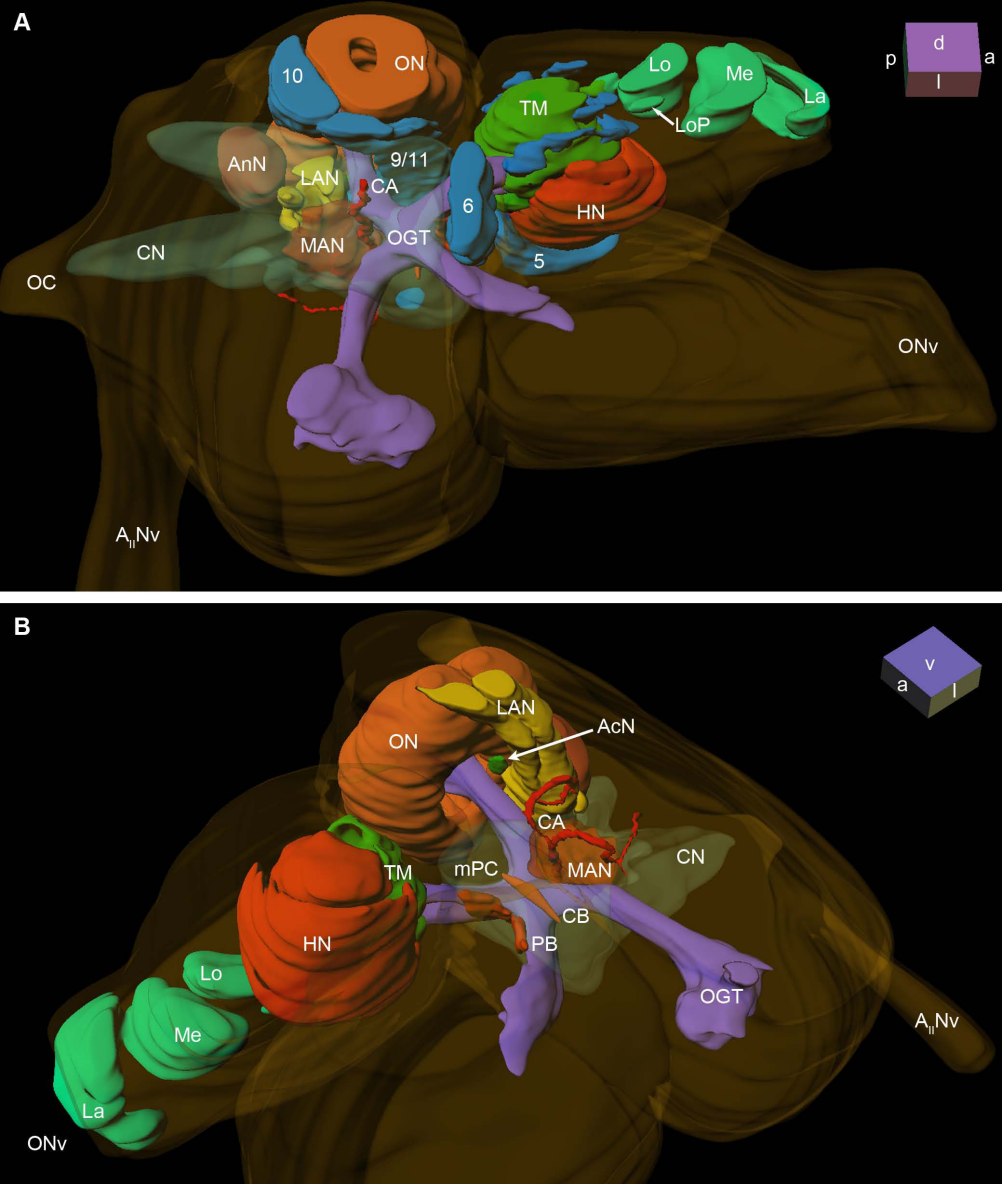
**Three dimensional brain reconstruction of *Birgus latro***. Only the left hemisphere of bilaterally paired neuropils and cell clusters is shown, when viewed from dorsal. The cell clusters shown in A, are left out in B, to display the ventral surface structure of neuropils and the neuropils of the medial protocerebrum. Numbers 5, 6, 10 and 9/11 (blue) identify cell clusters. Other abbreviations: A_II_Nv, antenna II nerve; AcN, accessory neuropil; AnN, antenna II neuropil; CA, cerebral artery; CB, central body neuropil; CN, columnar neuropil, HN, hemiellipsoid neuropil; La, lamina; LAN, lateral antenna I neuropil; Lo, lobula; LoP, lobula plate; Me, medulla; MAN, median antenna I neuropil; mPC, medial protocerebrum; OC, oesophageal connective; OGT, olfactory globular tract; ON, olfactory neuropil (olfactory lobe); PB, protocerebral bridge; TM, terminal medulla.

**Figure 8:**
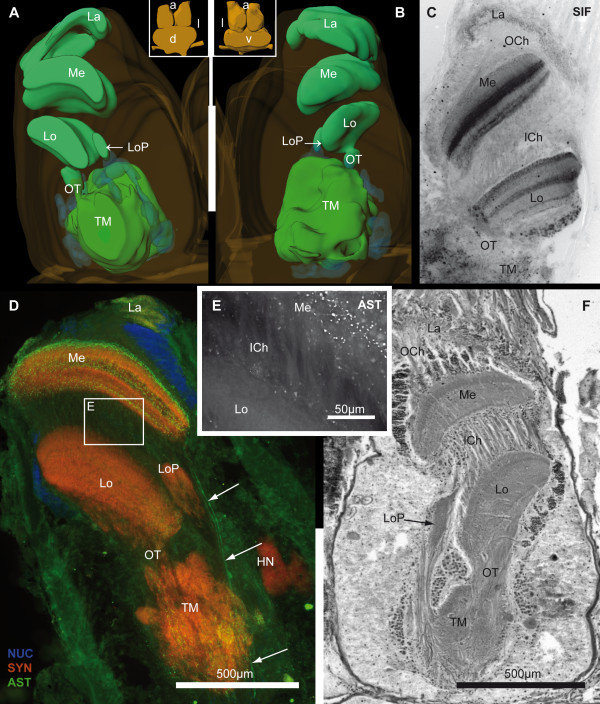
**The lateral protocerebrum - optic neuropils**. **A, B**: The detailed 3D-reconstruction of the left brain hemisphere is shown from dorsal and ventral (see inserted brain orientations). The corresponding cell clusters (blue) are displayed semitransparently. **C**: Inverted picture of a horizontal vibratome section labeled for SIFamide-like immunoreactivity (SIF; black). **D**: Horizontal vibratome section triple labeled against cell nuclei (NUC; blue), synapsin (SYN; red) and allatostatin-like (AST; green) immunoreactivity in higher magnification from Fig. 3A. The arrows mark several bypass fibers originating from the medulla (M). Based on the boxed area in D, the single ASTir-channel (white) is shown in higher magnification in **E **to visualize the crossed fibers of the inner optic chiasm (ICh). **F**: Silver impregnation of a microtome slice is shown in the same section plane as in D. Numbers 1 to 3 identify cell clusters. Other abbreviations: HN, hemiellipsoid neuropil; La, lamina; Lo, lobula; LoP, lobula plate; OCh, outer optic chiasm; OT, optic tract; TM, terminal medulla.

**Figure 9:**
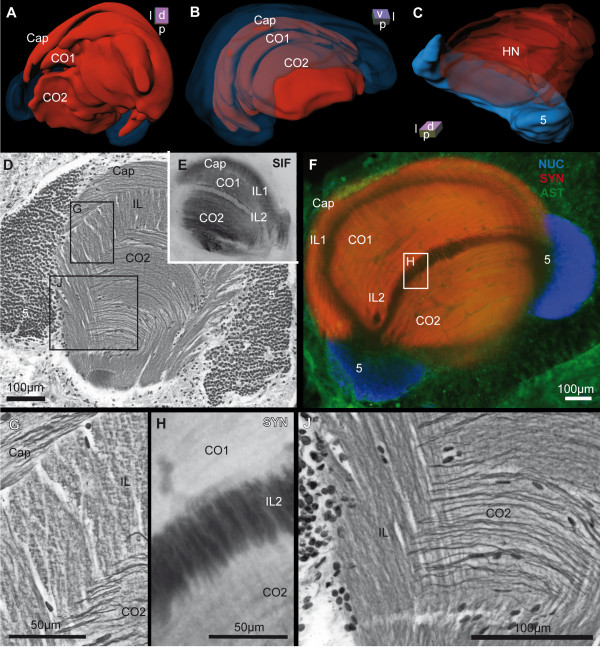
**The hemiellipsoid neuropil and its corresponding cell cluster**. **A to C**: 3-D reconstructions of the hemiellipsoid neuropil (HN) of the left brain hemisphere in different orientations. In A and B the cell cluster 5 and in C the HN is displayed semitransparently, to show the subjacent structures. **D**: Silver impregnation of a microtome slice is shown, and the boxed areas are pictured in higher magnification in **G **and **J**, making the orientation of fibers within the various layers visible. Note that in E, the horizontal vibratome section with immunohistochemical labeling against SIFamide (SIF; black) is obtained from another animal, but in a similar section plane. **F**: Horizontal vibratome section triple labeled against cell nuclei (NUC; blue), synapsin (SYN; red) and allatostatin-like (AST; green) immunoreactivity. The boxed area in F is illustrated in higher magnification in **H**, to present the synaptic interconnections within the second intermediate layer, in a single-channel view for anti-synapsin (white) immunoreactivity. Abbreviations: 5, cell cluster 5; Cap, Cap neuropil; CO1, outer or 1^st ^core neuropil; CO2, inner or 2^nd ^core neuropil; IL, intermediate layer; IL1 and IL2, outer or 1^st ^and inner or 2^nd ^intermediate layer (subunits of the IL).

**Figure 10:**
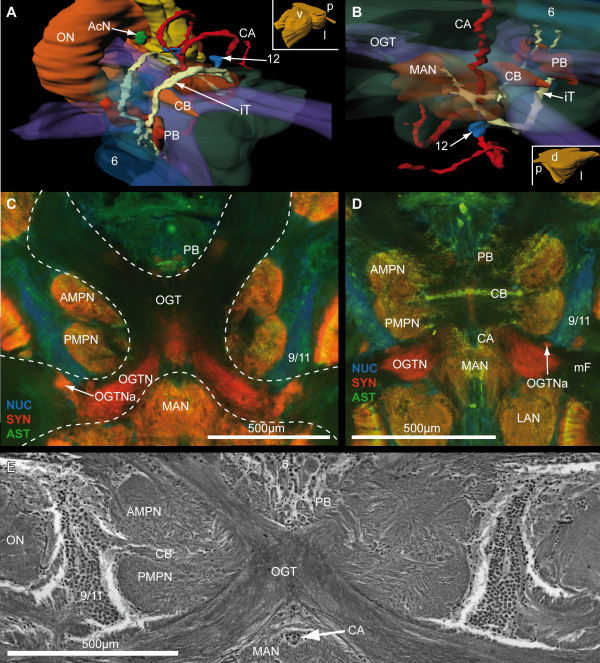
**The medial protocerebrum and the olfactory globular tract**. **A and B**: 3-D reconstruction protocerebral, deutocerebral neuropils and the olfactory globular tract in different orientation (see boxed figures). In A and B the cell cluster 6, the MAN, the OGT and the mPC fused with the CN are shown semitransparently, to make to visualize the subjacent structures. **C and D**: Horizontal vibratome section triple labeled against cell nuclei (NUC; blue), synapsin (SYN; red) and allatostatin-like (AST; green) immunoreactivity. In C, the negative print of the OGT is encircled by an interrupted line. **E**: A silver impregnation of a microtome slice is shown in a similar section plane. Arrowheads mark several neurons with strong ASTir. Numbers 6, 9/11 and 12 identify cell clusters. Other abbreviations: AcN, accessory neuropil; AMPN, anterior medial protocerebral neuropil; CA, cerebral artery; CB, central body neuropil; iT, interconnecting tract; LAN, lateral antenna I neuropil; MAN, median antenna I neuropil; mF, median foramen; OGT, olfactory globular tract, OGTN, OGT neuropil; OGTNa, accessory OGT neuropils; ON, olfactory neuropil; PB, protocerebral bridge neuropil; PMPN, posterior medial protocerebral neuropil.

**Figure 11:**
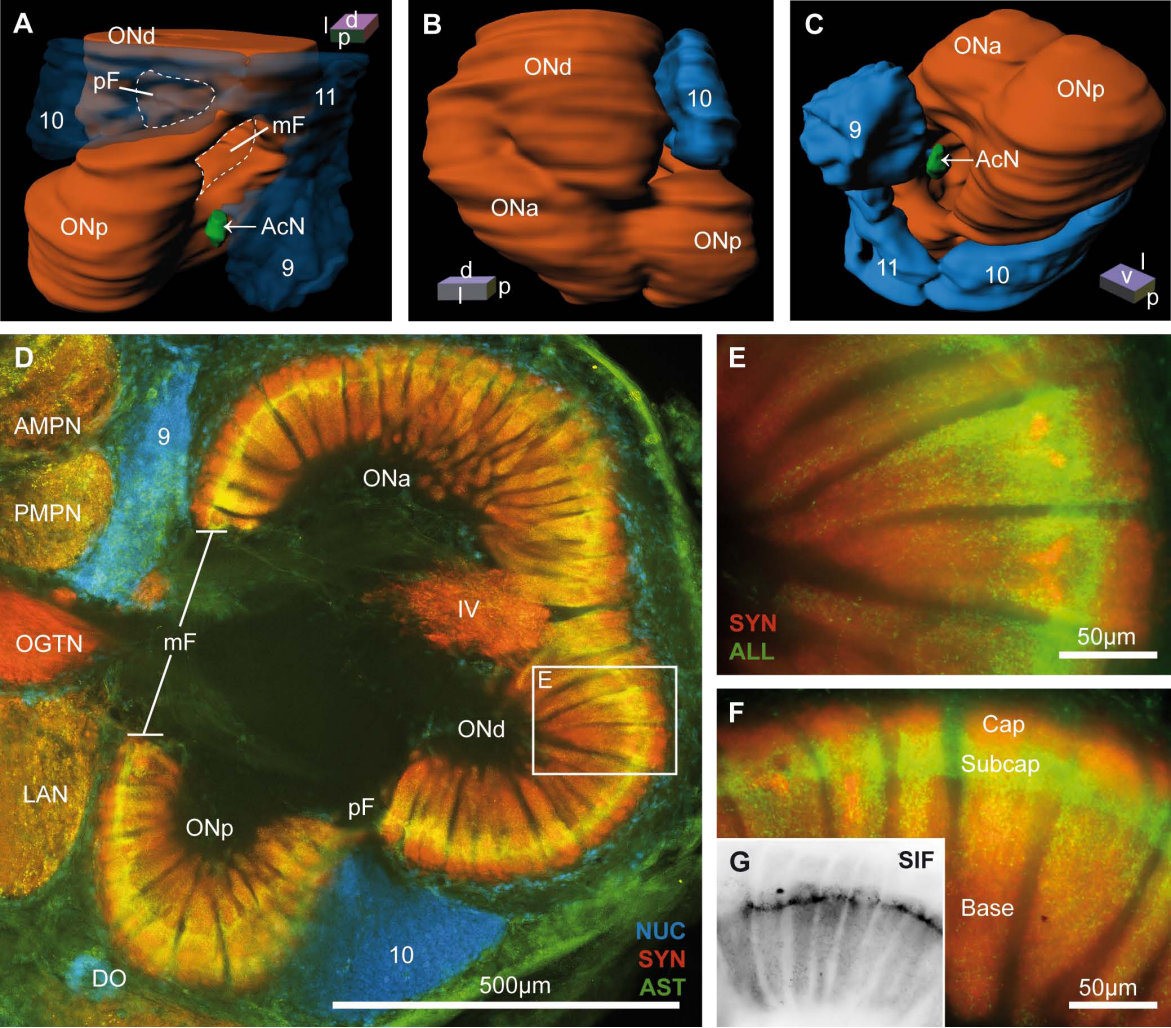
**The deutocerebral olfactory neuropil**. **A to C**: 3D-reconstruction of the olfactory and accessory neuropil in different orientations. In A, the cell clusters are shown semitranparently to visualize the subjacent structures. **D to F**: Horizontal vibratome section double or triple labeled against cell nuclei (NUC; blue), synapsin (SYN; red) and allatostatin-like (AST; green) immunoreactivity in higher magnification from Fig. 4C. The three subdivided regions of the glomeruli are shown in F (higher magnificated from Fig. 5C), marked with Cap, Subcap and Base. Note that in **G**, the horizontal vibratome section with immunohistochemical labeling against SIFamide (SIF; black) is obtained from another animal, but in a similar section plane like F. Roman numeral IV identifies an unstructured neuropil within the olfactory neuropil (ON). Numbers 9 to 11 identify cell clusters. Other abbreviations: AcN, accessory neuropil; AMPN, anterior medial protocerebral neuropil; mF, median foramen; DO, deutocerebral organ; OGTN(a), (accessory) olfactory globular tract neuropil; ONa, anterior; ONd, dorsal and ONp, posterior sublobes of the olfactory neuropil; pF, posterior foramen; PMPN, posterior medial protocerebral neuropil.

**Figure 12:**
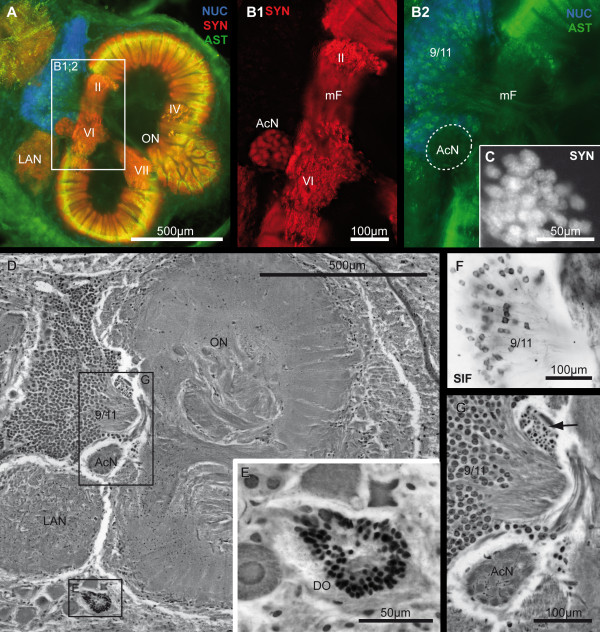
**The accessory neuropil and the deutocerebral organ**. Histological sections of cell cluster (9/11), the accessory neuropil (AcN) and the deutocerebral organ (DO) in detail. **A to C**: Horizontal vibratome triple labeled against cell nuclei (NUC; blue), synapsin (SYN; red/white) and allatostatin-like (AST; green) immunoreactivity in higher magnification from Fig. 5C. In **B1**, only the SYN-channel and in **B2**, the AST- and the NUC-channel are displayed separately. Note that in **F**, the horizontal vibratome section with immunohistochemical labeling against SIFamide (SIF; black) is obtained from another animal, but in a similar section plane like A to C. **D**: A silver impregnation of a microtome slice is shown in a similar section plane. E and G display higher magnifications of the boxed areas, shown in D. Roman numerals I, II, IV and V identify unstructured neuropils within the olfactory neuropil (ON). Other abbreviations: arrow, corresponding cell cluster to the AcN; LAN, lateral antenna I neuropil; mF, median foramen.

**Figure 13:**
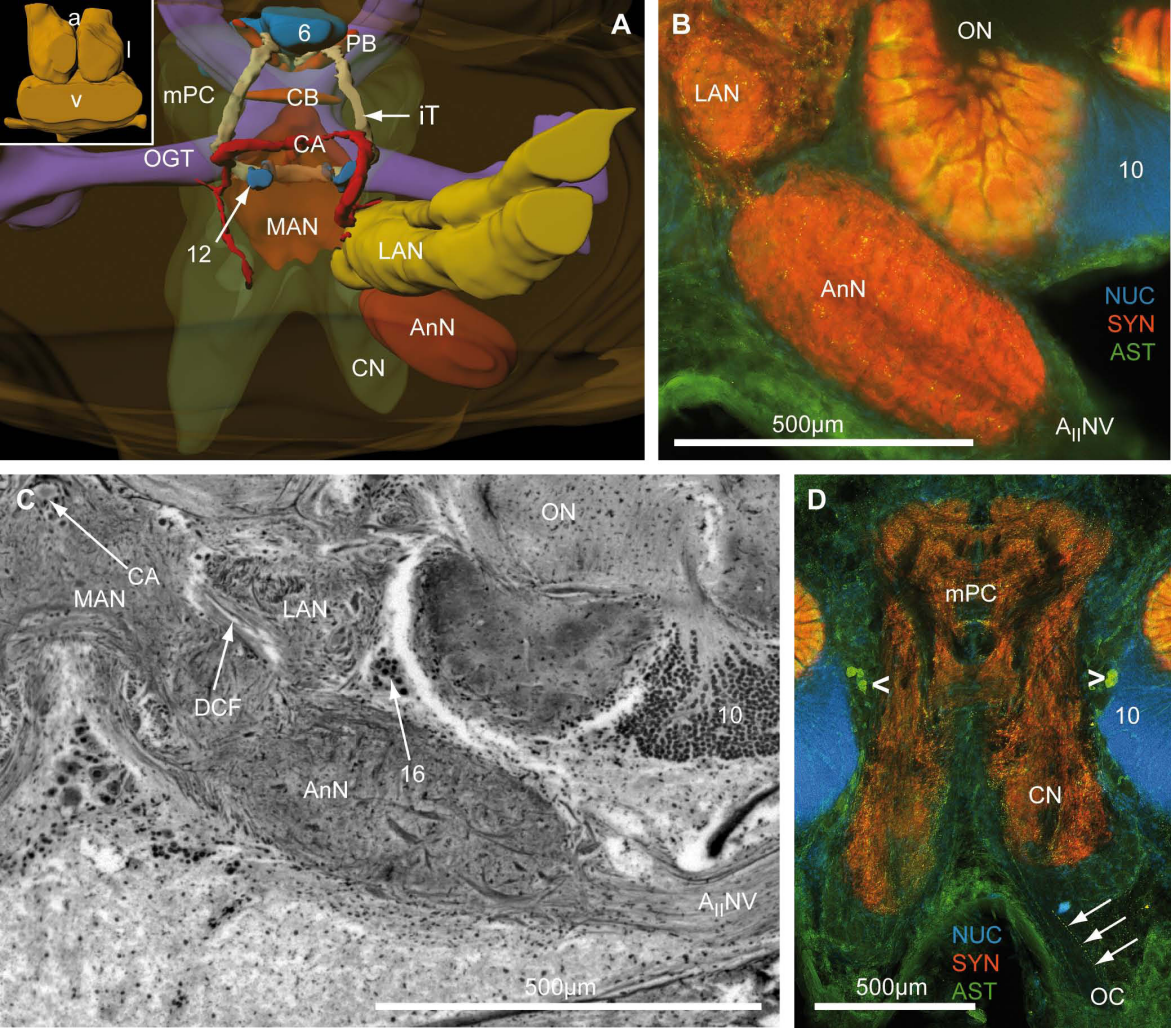
**Posterior Deutocerebrum and Tritocerebrum**. **A**: 3D-reconstruction of the mid-brain from ventral (see box). **B**: Horizontal vibratome section, triple labeled against cell nuclei (NUC; blue), synapsin (SYN; red) and allatostatin-like (AST; green) immunoreactivity in higher magnifications from Fig. 4A. **C**: A silver impregnation of a microtome slice is shown in a similar section plane. **D**: The fused columnar neuropil (CN) further dorsally with the same triple counter stains as in B. Numbers 6, 10 and 16 identify cell clusters. Other abbreviations: arrowheads: identifiable groups of single neurons showing ASTir; arrows: fibers with ASTir inserting the oesophageal commissure (OC); A_II_Nv, antenna II nerve; AnN, antenna II neuropil; CA, cerebral artery; CB, central body neuropil; DCF, deutocerebral commissural fibers; iT, interconnecting tract; LAN, lateral antenna I neuropil; MAN, median antenna I neuropil; mPC, medial protocerebrum; OGT, olfactory globular tract; ON, olfactory neuropil; PB, protocerebral bridge neuropil.

The brain of *B. latro *has an approximate size of 4 mm in length and 3 mm in width and its volume amounts to ca. 1.01 × 10^10 ^μm^3^ or ≈ 10 μl. In Fig. 3A, C, 4A, C, 5A, C, 6A and 6C, a silver-stained series of the brain is represented from dorsal to ventral. In Fig. 3B, D, 4B, D, 5B, D, 6B and 6D, a section series of the triple-labeling experiments of another animal's brain with nuclear counterstains (blue), anti-synapsin (red) and anti-allatostatin (green) immunohistochemistry is shown in corresponding section planes. The entire brain is surrounded by a tissue layer, the sheath, that exhibits unspecific labeling of allatostatin-like immunoreactivity (ASTir; Fig. 3, 4,5, 6 to the right) and is also heavily labeled in the silver impregnated preparation (Fig. 3, 4, 5, 6 to the left). From anterior to posterior, the brain is composed of the proto-, deuto- and tritocerebrum. The protocerebrum can be subdivided into an anterior lateral portion consisting of the bilaterally paired optic neuropils, as well as the lateral protocerebrum and the unpaired medial protocerebrum, with e.g. the protocerebral bridge neuropil (PB) and the central body neuropil (CB). More posteriorly, belonging to the deutocerebrum, the olfactory neuropils (ON), the accessory neuropils (AcN), the paired lateral (LAN) and the unpaired median (MAN) antenna I neuropils, and the columnar neuropil (CN) are located and posteriorly adjoined by the tritocerebral antenna II neuropils (AnN; Fig. 2, 3, 4, 5, 6). A three-dimensional (3D) reconstruction based on 89 silver impregnated cross sections is presented in Fig. 7 to show the spatial arrangement of these neuropils. The optic neuropils that receive direct visual input from the compound eyes *via *the optic nerve (ONv) are visible in the most dorsal sections (Fig. 3, 4), and display strong synapsin-immunoreactivity (SYNir). The optic neuropils are, from anterior/distal to posterior/proximal, the lamina (La), the medulla (Me), the lobula (Lo, synonymous with the medulla interna) and the lobula plate (LoP). The optic neuropils are connected to the terminal medulla (TM, synonymous with the medulla terminalis) and the hemiellipsoid neuropil (HN) *via *the optic tract (OT). These two neuropils together represent the lateral protocerebum. The HN (Fig. 3, 4, 5, 6, 8D, 9) displays strong SYNir and is associated with the goblet-shaped cell cluster (5) that contains the somata of densely packed interneurons [45]. The olfactory globular tract (OGT) links the deutocerebral olfactory and accessory neuropils (ON, AcN) and the lateral protocerebrum. It contains bundles of axons extending to the ipsilateral protocerebrum and also fibers that cross the midline towards the contralateral protocerebrum, thus forming a median chiasm (X; Fig. 2, 4, 7, 10C, D, E). Within the OGT, a further neuropil is present, displaying strong SYNir, the olfactory globular tract neuropil (OGTN; Fig. 4A, C, 10C, D, 11D). The OGT represents the major output pathway of the olfactory system [45-47] being composed of the axons of numerous olfactory projection neurons, which somata are located in a dorsoventrally extending cell cluster (10). The deutocerebral ON is the dominating neuropil within the brain and is targeted by the primary afferents of the olfactory sensory neurons of the first antenna (Ant I; antennule). The ON is tripartite and composed of more than 1300 so-called "olfactory glomeruli" (Fig. 2, 3, 4, 5, 6, 11D, E, F, G,). These structures, which show strong SYNir and ASTir, are arranged radially along the periphery of the ON. The absence of SYNir and the weak ASTir signal shows that the core of the latter is filled with bundles of fibrous material and does not contain any synaptic neuropils. The distinct accessory neuropil (AcN; Fig. 2, 5, 7B, 10A, 11A, C, 12A, B, C, D, G) is located medially to the ON where the OGT enters/exits the ON, close to the median foramen (mF). The AcN is much smaller than the ON and contains numerous spherical glomeruli that show a strong SYNir, but are almost devoid of ASTir (Fig. 12A, B, C). Anterior to the AcN, nuclear labeling reveals at least one, maybe two further compact cell cluster with densely packed nuclei. These (9/11) comprise the somata of local olfactory interneurons [45]. Subgroups of these neurons display both ASTir and SIFamide-like immunoreactivity (SIFir) in their cytoplasm (Fig. 11A, B1, B2, F). Sandeman et al. [45] distinguished between a dorsal (11) and a ventral (9) cell cluster in Astacura, Brachyura and Palinura. A distinct border inside the cluster (9/11) was not visible in *B. latro *(Fig. 11A, B, C), however, the axons of the interneurons in cell cluster (11) in the macrurans comprise the deutocerebral commissure and, apart from a small number of large olfactory interneurons [48] these project only to the AcN. The deutocerebral commissure is apparently absent in the brain of *Birgus latro*. Hence, in *B. latro *the cluster (11) may be very small given the tiny accessory neuropil, or even absent if this class of interneurons is no longer present (see discussion). Anterio-medially to the ON, the medial protocerebrum (mPC) can be subdivided into an anterior (AMPN) and a posterior medial protocerebral neuropil (PMPN). Nevertheless, these neuropils are fused in the dorsal most and ventral most sections, so that a separation is only obvious in the mid horizontal layers. Between the AMPN and the PMPN, an unpaired transverse, spindle-shaped neuropil displays strong ASTir and SYNir. This is the central body neuropil (CB; Fig. 4, 7B, 10) that extends across the midline. Further anteriorly to the AMPN, the protocerebral bridge neuropil (PB; Fig. 3C, D, 4C, D, 7B, 10, 13A) is visible, showing ASTir and SYNir. The lateral protocerebrum and the AMPN are connected *via *the protocerebral tract (PT; Fig. 2, 3C, D, 4D), which shows a weak ASTir signal and is located anteriorly to the AMPN. The cell cluster (6), located anteriorly to the AMPN, includes at least four neurons with somata diameters up to 50 μm among hundreds of neuronal somata with diameters of 5-18 μm. Many cells in cluster (6) display strong ASTir (Fig. 4A, C, 5A, 10C, D) and SIFir (data not shown here). Posteriorly to the PMPN, the paired lateral antenna I neuropils (LAN; Fig. 2, 4, 5, 6B, 7, 10A, D, 11D, 12A, D, 13A, B, C), which receive mechanosensory and non-aesthetasc chemosensory afferents from the ipsilateral first antennae, extend dorsoventrally across the brain. Similar to the LAN, the unpaired median antenna I neuropil (MAN; Fig. 2, 4, 7, 10) shows positive immunoreactivity for synapsin, allatostatin and SIFamide. The MAN is located posteriomedially to the PMPN on the midline and is dorsoventrally penetrated by the cerebral artery (CA; Fig. 2, 3, 7, 10, 13A). The nuclei of endothelial cells, as labeled by the nuclear marker, are arranged peripherally along the cerebral artery (Fig. 3A, C). The posteriormost neuropil of the brain is the tritocerebral antenna II neuropil (AnN; Fig. 2, 3C, D, 4, 7A, 13A, B, C), which has an ellipsoid shape and displays strong SYNir. The AnN receives the primary input from the second antenna and contains the synaptic fields of the efferent motorneurons of antenna II [45]. Further dorsally, the borders of the mPC, the MAN and the AnN become more indistinct and finally form a fused columnar neuropil (CN; Fig. 2, 3A, B, 7, 13A, D) with strong SYNir.

### Protocerebrum: The optic neuropils

The most anterior part of the brain is represented by the stacked optic neuropils, the lamina (La), the medulla (Me), the lobula (Lo) and the lobula plate (LoP), which process the primary optic input from the compound eyes. In *B. latro*, these neuropils are located close to the median part of the brain, whereas in other decapods they are enclosed in the eyestalks (compare [43]). As a consequence, the retinula cell axons that extend from the ommatidia into the lamina and medulla may measure up to 10 mm or more [43]. Immunolocalization of allatostatin and SIFamide reveals differences in the distribution of these neuropeptides. For example, the lamina and the medulla show stronger ASTir than the lobula, whereas the lobula and the medulla display stronger SIFir than the lamina (Fig. 8). The lamina shows weak SYNir, moderate SIFir, and strong ASTir, and is composed of a thin synaptic layer shaped like a cap, which is distally covered by a layer of cell bodies (cell cluster (1), according to Sandeman et al. [45]). The lamina is connected to the medulla by crossing fibers that form the first or outer optic chiasm (OCh). In addition, the ellipsoid medulla possesses multiple nervous connections to different other target structures, namely the lobula, the lobula plate *via *the optic tract (OT) and also a third connection where fibers display ASTir and bypass the terminal medulla to target the medial protocerebrum (Fig. 3A, 8D, 14A). Furthermore, immunohistochemistry in cross sections shows that the medulla has a characteristic organization with retinotopic layers (Fig. 3A, C, 8C, D), similar to the lamina and the lobula. Altogether, three layers can be differentiated in SIFir and ASTir preparations, of which the anteriormost is the most prominent layer. Between the lamina and the medulla, the interconnecting fibers are surrounded by many cell bodies (cell cluster (2), according to Sandeman et al. [45]). The lobula is located posteriorly to the medulla from which it receives retinotopic input *via *crossed fibers that form the inner optic chiasm (ICh; according to Sztarker et al. [49]; Fig. 2, 8C, E, F). As mentioned before, the lobula possesses several parallel arranged layers showing SIFir and weak ASTir. This neuropil is strongly innervated by a group of visual neurons located in an anteriolateral position with regard to the lobula (cell cluster (3) according to Sandeman et al. [45], Fig. 8D, F). The lobula is closely associated with the terminal medulla (TM), so that the optic tract (OT) is quite short. As in previous studies of brachyuran and anomuran decapods [40,49], *B. latro *in addition to the lamina, the medulla and the lobula, features a fourth optic neuropil, the lobula plate neuropil. This rather small neuropil is interposed between the medulla and the terminal medulla of the lateral protocerebrum and displays SYNir, SIFir and weak ASTir. In the silver impregnated sections, the lobula plate seems to be entirely wrapped by fibers that extend between the medulla and the terminal medulla (Fig. 2, 8F). From the eyestalk neuropils, various optic pathways proceed to the lateral and medial protocerebrum.

**Figure 14:**
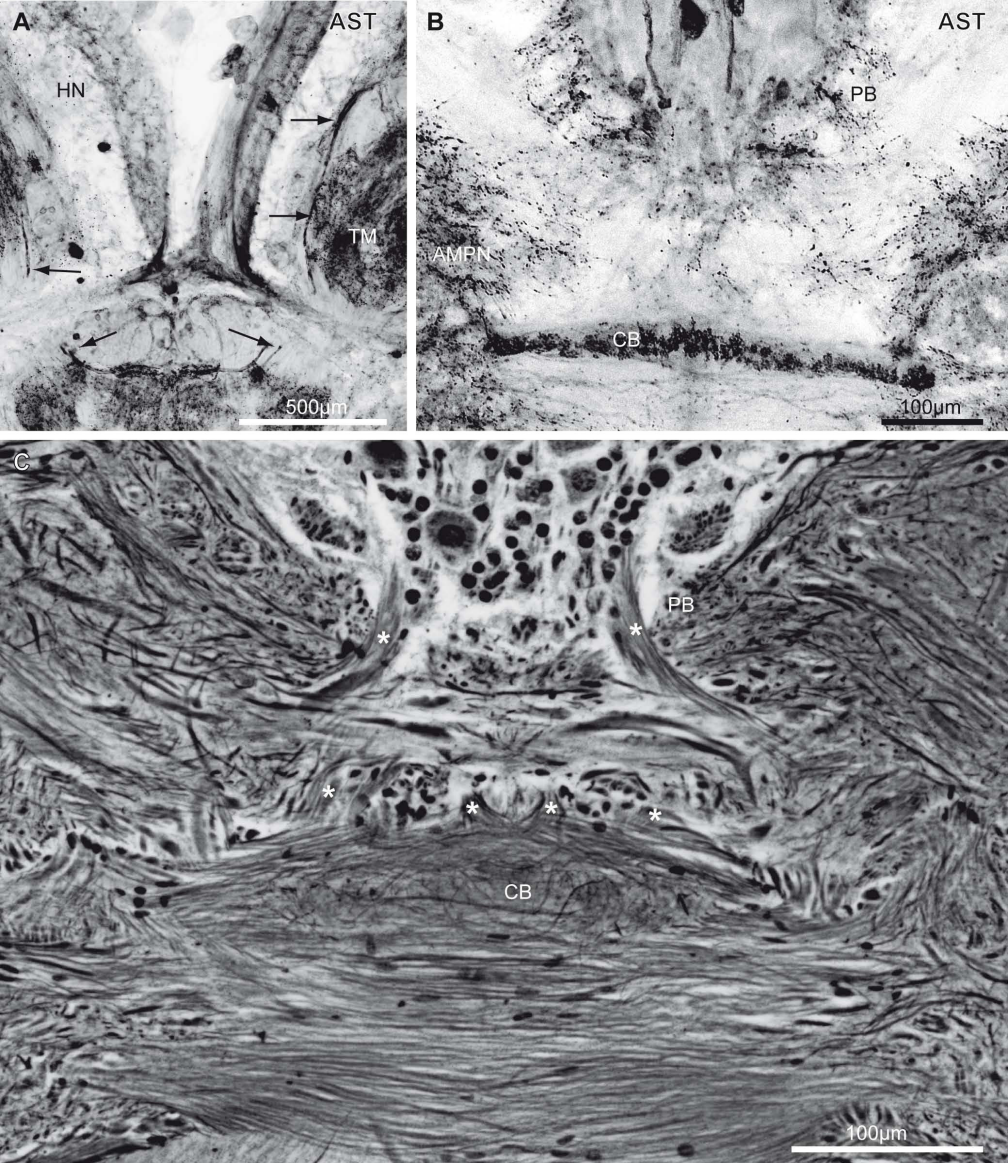
**Bypass fibers, the protocerebral bridge neuropil and the central body neuropil showing allatostatin-like immunoreactivity (AST; green)**. **A**: Inverted single channel picture of a horizontal vibratome section, allatostatin-like (AST; black) immunoreactivity in higher magnifications from Fig. 3A. The arrows identify possibly related fibers, bypassing the protocerebral tract and showing clear ASTir. **B**: Inverted single channel picture of a horizontal vibratome section, allatostatin-like (AST; black) immunoreactivity in the protocerebral bridge neuropil (PB) and the central body neuropil (CB) in higher magnifications from Fig. 4C. **C**: A silver impregnation of a microtome slice is shown in a similar section plane and higher magnification. Asterisks label several identifiable fiber bundles connecting the PB with the CB. Other abbreviations: AMPN, anterior medial protocerebral neuropil HN, hemiellipsoid neuropil; TM, terminal medulla.

### Protocerebrum: The lateral protocerebrum

Between the optic neuropils, two closely associated neuropils are present, the hemiellipsoid neuropil (HN) and the terminal medulla (TM, medulla terminalis), which together constitute the lateral protocerebrum. As mentioned above, the latter is connected to the optic neuropils *via *the optic tract. The lobula is an ontogenetic derivative of the TM, and thus also belongs to the lateral protocerebrum [50]. The TM and the HN together form a large sphere of approximately 900 μm in diameter, in which the TM is located more dorsoposteriorly and the HN ventroanteriorly. Posteriorly, the lateral protocerebrum is connected to the medial protocerebrum *via *the protocerebral tract (PT; Fig. 2, 3C, D, 4D) that includes the OGT that houses ascending fibers from the deutocerebral accessory and olfactory neuropil. In contrast to the HN, the TM is not geometrically arranged [45,51] and shows SYNir, and strong ASTir and SIFir. On the one hand, the medial neuropil regions of the TM are innervated by the lobula plate and the lobula *via *the optic tract, and on the other hand they receive strong input from the olfactory pathways of the deutocerebrum *via *the OGT (Fig. 2). In the 3 D reconstruction of the brain, several groups of cell bodies can be seen to be arranged like a ring around the TM (Fig. 7A, 8A, B). The hemiellipsoid neuropil with an average volume of 1.91 × 10^8 ^μm^3^ (Table. 1), has a highly complex type of onionskin-like organization (Fig. 3C, D, 4, 5, 6, 7, 9), with three distinct synaptic layers. Similar to the brain of the coenobitid hermit crab *Coenobita clypeatus *(Herbst, 1791; Anomura, Coenobitidae; [40]), a near relative of *B. latro*, these layers of synaptic fields form two inner core neuropils (CO1, CO2) and a peripheral cap neuropil (Cap; Fig. 9), which show strong SYNir and SIFir. In addition to few longitudinally extending axons, these layers also contain numerous fibers, progressing transversely in parallel to the anterior margin of the HN (Fig. 9D, G, J). When viewed in horizontal sections, these fibers form a grid-like structure (Fig. 9G, J). The subunits of the dorsally fused HN are ventrally separated by two clear intermediate layers (IL1, IL2; Fig. 9E, F), formed by several parallel axons that are arranged longitudinally. Higher magnification reveals that there are synaptic interconnections between Cap and CO1, and between CO1 and CO2 among the intermediate layers (Fig. 9G, H). Within the SYNir labeled neuropils, several gaps and nuclei appear, belonging presumably to the vasculature. The HN surmounts a large goblet-shaped cell cluster (5) of densely packed, small interneurons and is invaded by their axons from the ventral periphery (Fig. 9A, B, C, D, F). Both TM and HN are strongly innervated by numerous axons of deutocerebral projection neurons, which somata lie in the cell cluster (10) posteriolaterally adjacent to the olfactory neuropil. At the mid level of the horizontal sections, a prominent tract is visible, the olfactory globular tract (OGT; Fig. 4B, D, 7, 10), that, as mentioned above, interconnects the deutocerebral olfactory neuropils with the neuropils of the lateral protocerebrum. Its enormous arms, formed by bundles of the very small diameter axons of the projection neurons with their somata in cell cluster (10), have a diameter up to 160 μm in *B. latro*. In sections labeled by immunohistochemistry, the OGT appears as a sort of negative print with the markers that we used (Fig. 4A, C). From the olfactory neuropil, the OGT projects in an anterio-medial direction, where it touches its contralateral counterpart and forming a chiasm (X, Fig. 4A, D, 10C, E) slightly dorsal of the CB. As in other decapods [46,51] the nervous fibers emerging from the ON partially bifurcate at the chiasm and ascends towards the ipsilateral as well as to the contralateral protocerebral neuropils inside the eyestalks. There, the OGT inserts posteriorly at the transition between TM and HN and sends branches into both neuropils (Fig. 2, 4C, D). Unfortunately, we could not trace the course of these branches in detail with the methods applied.

### Protocerebrum: The medial protocerebrum

The medial protocerebrum (mPC) is medially positioned between the lateral protocerebrum and the deutocerebrum, and encloses the portion of the OGT forming its chiasm. In a few horizontal sections (Fig. 3C, D, 4), the division of the mPC by the central body neuropil (CB) in an anterior part (AMPN) and a posterior part, the posterior medial protocerebrum (PMPN), as mentioned above, becomes visible. The central complex in the protocerebrum is composed of the protocerebral bridge neuropil (PB), an anterior cell cluster (6) and the central body neuropil (CB). As is known from previous studies on Decapoda, the PB and mainly the CB are well equipped with diverse neuroactive substances (e.g. [52,53] and in *B. latro *shows strong SIFir (not shown) and ASTir (Fig. 3A, C, 4A, C, 10C, D, 14A, B). Most anteriorly, neuronal somata with various diameters (4-40 μm), are housed in the cell cluster (6), which broadens from ventral to dorsal (Fig. 7A, 10A, B, 13A). Utting and co-workers [53] described at least five different types of neurons within this cell cluster in the crayfish. In *B. latro*, Many cluster (6) somata and their fibers show strong ASTir (Fig. 3A, C, 4A, C, 5A, 10C, D). The PB is V-shaped, when viewed in horizontal sections, and its bilateral, symmetrical neuropil compartments fuse at the midline (Fig. 7B, 10A, B), posteriorly to the cell cluster (6). In further dorsal sections, few parallel transversally arranged fibers with clear ASTir are present, the ends of which point to the visible ends of the bypass fibers (Fig. 14A), that originate from the medulla (Fig. 8D). These fibers form an additional connection between the optic neuropils and the medial protocerebrum to the OGT and OT, and possibly form a chiasm anterior to the mPC. In addition, bundles of axons from projection neurons of the lateral protocerebrum, ending at the contralateral part of the PMPN, progress anteriorly and ventrally along the CB [53]. The cigar-shaped central body is an unpaired midline neuropil located centrally within the mPC and marks the boundary between the AMPN and the PMPN. In a few sections, it becomes visible by immunohistochemical labeling with ASTir (Fig. 4A, C, 10D), and it is well detectable in the silver impregnated sections (Fig. 10E, 14C). Several fiber bundles connecting the PB with the CB become visible in silver impregnated sections with higher magnification (asterisks in Fig. 14C). These bundles possibly correspond with the W-, X-, Y- and Z-fiber bundles which have been previously described by Utting and co-workers [53] in the crayfish *Cherax destructor*. The CB is typically located, slightly ventral to the chiasm of the OGT.

### Deutocerebrum: The olfactory neuropils

The most prominent neuropil of the *B. latro *brain is the olfactory neuropil (ON) with an average volume of 3.75 × 10^9 ^μm^3^, both hemispheres accounting for a percentage of 26.3% of the total neuropil volume (Table. 2). The ON receives chemosensory input from olfactory sensory neurons on the ipsilateral first antenna *via *the antenna I nerve (A_I_Nv). Their axons invade the ON from an anterio-ventral direction (data not shown here). Two large cell clusters are associated with the ON, the cell cluster (10) with about 160,000 projection neuron somata, which have an average nuclear diameter of 5.43 μm, and the smaller interneuron cell cluster (9/11), which contains circa 90,000 somata. The most ventral cell bodies in the latter cluster have a smaller diameter than the dorsal ones, which is one of the characteristics to distinguish cells of cluster (9), ventrally from those of cluster (11), dorsally. Subpopulations of cell cluster (9/11) show strong cytoplasmatic ASTir and SIFir (Fig. 12A, B2, F). In silver sections, the transition from cluster (9) to (11) is indistinct. The ON is subdivided into three partitions, an anterio-ventral (ONa), a dorsolateral (ONd) and a posterior-ventral sublobe (ONp; Fig. 11A, B, C, D). Each ON houses ca. 1,300 peripherally arranged olfactory glomeruli on average (Table. 2), each showing a typical pattern of strong SYNir and ASTir/SIFir. The olfactory glomeruli combine a conical and columnar shape, and are subdivided into a so-called cap, subcap, and base region. The ASTir and SIFir are stereotypically pronounced in the subcap region (Fig. 11D, E, F, G). The center of the ON is devoid of immunoreactivity except weak ASTir, but in silver stained cross-sections it becomes clear that various fiber bundles, carrying e.g. the major neurites of local interneurons in cluster (9/11) that target the base of the glomeruli, are present. Projection fibers of the OGT and approaching axons of the cell cluster (9/11) penetrate through the median foramen (mF) into the ON, a gap in the radial array of glomeruli. On the opposite margin of the ON, the neurites of cell cluster (10) enter the lobe *via *the posterior foramen (pF; Fig. 2, 11A, D). At the margin of these foramina and at the transition zones between the sublobes (ONa, ONp and ONd), several unstructured, non-glomerular synaptic fields are interspersed between the palisade-like array of olfactory glomeruli. These unstructured neuropils that display SYNir, ASTir and SIFir extend partly into the center of the ON (Roman numerals I-VI, from dorsal clockwise in the right hemisphere and anti-clockwise in the left hemisphere; Fig. 3A, 4A, C, 5C, 6A, 11D) and display an identical arrangement in both hemispheres. Six of these unstructured neuropils also occur in *C. clypeatus *in a corresponding arrangement (called A-F in Harzsch and Hansson [40]). Similar to *Coenobita clypeatus *[40], strong SYNir, but not any ASTir is present within the OGT posterior to its chiasm (Fig. 4A, C, 10C, D). These synaptic fields belong to the olfactory globular tract neuropil (OGTN). This neuropil contains at least two associated structures more distally, the accessory OGT neuropils (OGTNa). At higher magnification, these neuropils seem to have connections with the PMPN as well as with the deutocerebral MAN and LAN (Fig. 10C). Medially, adjacent to the ON and slightly ventral to the exit of the OGT, a comparatively small neuropil with an average volume of only 500,000 μm^3^ is present, the accessory neuropil AcN (Fig. 2, 5, 11A, C, 12A, B, C, D, G). This means, it is more than seven hundred times smaller than the ON. Similarly to *C. clypeatus *[40], the AcN has a spherical shape and is composed of circa 100 almost spherical glomeruli with a diameter of 10-16 μm (Fig. 12A, B, C). These glomeruli show strong SYNir, but weak ASTir and SIFir. In silver-stained cross sections, further anteriorly to the AcN, a small distinct cell cluster adjacent to cell cluster (9/11) is located. Considering the course of the fibers, which target the AcN (Fig. 12G), we suggest it to be a cluster of interneurons. Furthermore, an aggregation of approximately 550 cell somata (Table. 1) that form a hollow sphere are located posteriorly and slightly medially of the ON. This is the deutocerebral organ [54] (DO; Fig. 2, 4C, 5A, B, D, 11D, 12D, E; compare reviews [55-58]). Its small cell somata, as labeled with the nuclear marker, have an either spherical or elongated shape.

### Deutocerebrum: Other neuropils

Posterior to the mPC and flanked by the bilaterally paired OGTN, the unpaired median antenna I neuropil (MAN; Fig. 4, 7, 10, 13A, C) is located. It shows SYNir, strong ASTir and SIFir (not shown) in its center and has an almost spherical shape (Fig. 2, 10A, B, 13A). Ventral to the OGT, the MAN is connected to the PMPN *via *fibers showing ASTir (Fig. 10D). Further dorsally, distinct neuropil boundaries disappear and the MAN seems to be fused with protocerebral and tritocerebral neuropils as well, forming the columnar neuropil (CN). In silver-impregnated cross-sections, nervous fibers can be traced those extend dorsally from the PB towards the MAN, form a chiasm anterior to the MAN, and bypass the OGT ventrally (Fig. 10A, B). These fibers connect the medial protocerebrum with the deutocerebrum and are here named the interconnecting tract (iT). The somata associated with these fibers are grouped in cell cluster (12) anterioventral to the MAN and in cell cluster (6) anterior to the PB (Fig. 10A, B, 13A). Further posteriorly, deutocerebral commissural Fibers (DCF) are visible in silver-stained cross-sections (Fig. 4D, 13C), linking the cell cluster (16) across the MAN. The MAN is anteriorly penetrated by the dorsoventrally extending cerebral artery (CA; Fig. 2, 3A, B, C, 7, 10, 13A, C). The cerebral artery bifurcates immediately ventral to the ON. Its branches are bent dorso-posteriorly towards the lateral antenna I neuropils, where the capillary ramification of the CA become untraceable (see Fig. 10A, B). It has been shown in different decapod species that the MAN receives multimodal inputs from the first antenna, namely descending interneurons related to the statocysts, branches of the antenna I motorneurons and mechanosensory afferents from sensilla of the antennal base as well [45,59]. Posterio-laterally to the MAN, the LAN displays SYNir, ASTir and SIFir. This neuropil is arranged in two more or less distinct elongated columns that fuse at the level of the OGT (Fig. 10A, D). The LAN extends dorsoventrally and fuses with the CN dorsally.

### Tritocerebrum

The tritocerebrum is represented by the barrel-shaped antenna II neuropil (AnN), lying posteriorly adjacent to the LAN and ON. It processes afferent input from the second ipsilateral antenna and provides neuromuscular efferent output *via *the antenna II nerve (A_II_Nv). In cross-sections, labeled by immunohistochemistry, the AnN shows clear SYNir and ASTir. A stratified neuropil arrangement of the AnN like e.g. in *Pagurus bernhardus *(Krieger unpublished data) is indistinct in *B. latro *(Fig. 13B, C). Further dorsally, the AnN seems to be fused with the MAN and the mPC, forming the amorphous columnar neuropil (CN), which points posteriorly towards the oesophageal connective (OC). The tritocerebral tegumentary neuropil (TN) is not visible as a distinct structure in our data, but the tegumentary nerve (TNv) is present dorsally in several silver-impregnated cross-sections (Fig. 3B). The TNv extends towards the columnar neuropil, where the location of the TN is assumed (see [45,47]).

## Discussion

### The optic neuropils and visual abilities

In many respects the layout of the brain and general arrangement of neuropils in *B. latro *is quite similar to that in other decapods [43], specifically to that in another coenobitid, *Coenobita clypeatus *[40]. One major difference concerns the eyestalk neuropils, which in *B. latro *are shifted more proximally towards the brain so that the brain is longer than broad. This is also the case in a few other decapods species, e.g. *Callianassa australiensis *Dana, 1852 (Thallassinida) and *Petrolisthes lamarckii *Leach, 1820 (Anomura) [43,47]. The coconut crab features prominent eyestalks and fully developed compound eyes (Fig. 1) similar to other coenobitids. In fact, the retina is displaced many millimeters away from the brain. In other decapods, the strategy is to have the optic neuropils close to the eye so that retinula axons can be short but instead the protocerebral tract linking the optic neuropils and the lateral protocerebrum to the medial brain has to be long. On the contrary, *B. latro *has long retinula axons so that the protocerebral tract is very short. The functional role of this arrangement is still unresolved. Nevertheless, the neurochemistry and architecture of the three columnar optic neuropils in *B. latro *closely resemble that of other decapods [40,49,52,60] so that it is fair to assume that the visual analysis capacities of this creature matches those of other decapods. There is evidence that many terrestrial crustaceans, specifically members of the Brachyura have evolved good visual orientation abilities on land [5,61,62]. The visual system of fiddler crabs (Brachyura) is remarkable for its tuning to master the visual world of inter-tidal mud flats [62,63]. Their eyes are mounted on long vertical stalks and they have a panoramic visual field and a pronounced equatorial acute zone for vertical resolving power. Future anatomical studies on the ommatidia's optic apparatus will reveal which kind of adaptations have evolved in the *B. latro *eye to compensate for the different optic features of air *versus *water.

### The central olfactory pathway and associated structures

As mentioned before, *B. latro *has established a sense of aerial olfaction and is therefore capable to detect food sources from long distances [37], as was also suggested for *Coenobita clypeatus *[40]. In Crustacea, the olfactory stimuli are detected by olfactory sensory neurons housed within the aesthetascs on the lateral flagellum of the bilaterally paired first antennae [41,42,64]. The aesthetascs of *B. latro *are short and blunt and arranged in ordered rows along the ventral side of the lateral flagella [37,65]. The behavioral findings on the sense of aerial olfaction in this animal are mirrored in the architecture of the central olfactory pathway as its central components, the olfactory neuropils and the hemiellipsoid neuropils, are greatly enlarged compared to other decapods. This feature has already been observed in a close relative of *B. latro*, the land hermit crab *Coenobita clypeatus *[40]. Together, these two neuropils of the central olfactory pathway account for about 40% of the total neuropil volume (Table. 1). Olfaction can thus be suspected to be the major sensory modality that these brains process. The volume of the olfactory neuropils (AcN and ON) of freshwater crayfish (*C. destructor *and *P. clarkii*) and also the American lobster *Homarus americanus *represents 30-40% of the median brain volume [66,67]. Because these authors refer to the volume of the whole brain, not the neuropil volume, and because they do not include the volume of the optic ganglia into these calculations, it is difficult to compare these data with ours. Hanström [68] also conducted volumetric studies of several decapod crustaceans' brains and of other arthropods. His study is based on section series stained by hematoxylin and eosin. A comparison of absolute volumetric values with our data is difficult because Hanström [68] examined different species than we did and because he used a different set of histological and analytical methods which nevertheless was groundbreaking for that time. In the decapod species that he studied he reported the olfactory centers to take up between roughly 20% and 30% of the entire brain volume. For example, in the porcellanid crab *Petrolisthes cinctipes *Randall, 1840, the ("*Riechzentren*") olfactory centers amount to 23.9% of the whole brain volume, but the area of synaptic neuropil is presumably smaller and it is also unclear whether or not the author included the hemiellipsoid bodies as ("*Riechzentrum*") olfactory center.

The afferents from the first antennae enter the brain *via *the antenna I nerves from the ventral side and form a dense plexus that surrounds the entire ON. It is well known that in many decapods the primary afferents penetrate into and cross the entire olfactory glomeruli in a centripetal course from this plexus [69-71]. The olfactory glomeruli are arranged in a radial array around the periphery of the olfactory neuropil and are subdivided into a cap-, a base- and a subcap region [72,73], of which the latter shows strong ASTir in *B. latro*. From *Cherax destructor *Clark, 1936 is known that the subcap region of the olfactory glomeruli is interconnected with the ipsilateral accessory neuropil *via *neurites of olfactory interneurons that have their somata in cell cluster (9/11) [74]. We could not verify this connection with our methods, but in cluster (9/11) the somata of interneurons are present that show strong ASTir (Fig. 12A, B2), and the axons which project into the ON and most likely are the source of ASTir in the subcap of the glomeruli.

Another conspicuous feature of *B. latro *is the subdivision of its olfactory neuropil into three sublobes (Fig. 11; ONa, ONp and ONd), which seems to be unique among the anomuran decapods. In *C. clypeatus*, the olfactory neuropil is composed of two sublobes only, whereas most other decapods have only one lobe. This tripartition might be a primitive pre-stage of a cortical folding to increase the surface area, and thus offering a larger contact area between the enveloping plexus of the antennal nerves and the olfactory glomeruli. We estimate that this shape increases the surface area up to 30-40%, in comparison to a spherical body of the same volume. Furthermore, in combination with the elongate shape of the glomeruli, the subdivision into lobes may be another measure to economically increase the packing density of units in the system. Along these lines, the tendency to form sublobes observed in *Coenobita clypeatus *as well [40] may be an evolutionary precursor to the situation in *B. latro*.

In addition to the distinct olfactory glomeruli, the olfactory neuropil in *Birgus *contains six unstructured synaptic regions close to the posterior and medial foramina and at the transition from one sublobe to another. These unstructured neuropils, which interrupt the regular array of glomeruli and extend towards the center of the olfactory neuropil (Fig. 3A, 4A, C, 5C, 6A, 11D), are labelled by roman numerals I-VI. The function of these neuropils remains unclear. One hypothesis is that these may be precursor structures of the glomeruli. Previous studies in the American lobster *Homarus americanus *Weber, 1795 have, however, shown that the number of olfactory glomeruli is almost stable in adult specimens [75]. From developmental studies in *Cherax destructor *is known that the number of glomeruli is set in early development and that the synaptic area increases with growth (Sandeman R. unpublished). The unstructured neuropils thus may provide additional synaptic target areas for increasing numbers of afferent inputs without having to add extra glomeruli with growth. Developmental studies will have to show if this is also the case in Coenobitidae.

Whereas the olfactory lobes are well elaborated, the AcN is comparatively small in size and shows only weak ASTir. The AcN is known to receive higher-order multimodal inputs but no primary afferents in other decapods [46]. The small AcN present in *B. latro *is typical for Brachyura and Anomura, whereas e.g. in crayfish and spiny lobster the size of this neuropil may equal or even exceed that of the olfactory neuropils [45]. According to Sandeman et al. [43] and Sandeman and Scholtz [47], small accessory neuropils are a synapomorphic feature for the Meiura (Anomura+Brachyura; [76]). In *B. latro*, the silver impregnated sections revealed a cluster housing extremely small cells adjacent to cluster (9/11) that send their axons into the AcN. An important input to the large AcNs in *Cherax destructor *enters along axons of interneurons that have their cell bodies dorsally in cluster (11). These interneurons have unilateral inputs from various areas of the brain but their axons decussate in the deutocerebral commissure and project bilaterally to the two AcNs [77]. The deutocerebral commissure is not identifiable in *Birgus latro*, implying that with the decrease in size and importance of the AcN, the need to bilateralize the inputs from the brain to these areas is no longer necessary as in the astacurans. In *Cherax destructor *both physiological and anatomical evidence indicate a connection between the axons of the projection neurons and large serotonergic neurons, the dorsal giant neurons (DGN), that project to all the glomeruli in both the AcN and ON in these animals [77,78]. A DGN homologue in *Birgus latro *has yet to be found.

The olfactory neuropil is connected with the hemiellipsoid neuropil and the terminal medulla of the ipsilateral as well as the contralateral protocerebrum [74,79]. Sullivan and Beltz [46] reported that in three species of the Astacidea Latreille, 1802, ascending projection neurons from the olfactory neuropil innervated primarily the terminal medulla, whereas the projection neurons of the accessory neuropil targeted exclusively the hemiellipsoid body. Although our methods did not allow us to analyze these connections in *B. latro *in such detail, it seems unlikely that the small accessory neuropil would provide the sole input to the giant hemiellipsoid neuropil, but that instead input from the olfactory neuropils must be dominant. The lateral protocerebrum also receives input from the lateral and median antenna I neuropils, which process mechanosensory, non-aesthetasc chemosensory and statocyst input [45,71]. Therefore, it has been suggested that the neuropils of the lateral protocerebrum have an integrating function for the multimodal mechanosensory, optic and olfactory inputs that they receive (reviews [42,80]).

### Comparison to other crustaceans, functional implications and neuroethology

Among the marine and terrestrial Meiura (including anomuran and brachyuran crabs) studied so far (referring to [44]; Table. 2), *Birgus latro*, in absolute terms, has the largest olfactory neuropils, the largest olfactory glomeruli, and the highest number of olfactory glomeruli. Furthermore, the study by Harms 1932 [7] indicates that these animals also rank highly concerning the number of aesthetascs but it is difficult to extract which methods for counting this author used and what the size of the animals was that he examined. We estimate an aesthetasc number of 1,600 to 1,800 per lateral flagellum of an adult animal's first antenna, which is more than twice the number counted by Harms. Due to the fact that the aesthetasc number increases with growth in crayfish [44], we may assume that Harms examined a younger specimen than we did. Concerning absolute ON volume and glomerular numbers, the coconut crab together with other large crustaceans, spiny lobsters of the genera *Jasus *and *Panulirus *ranks among the top three of the decapods that have been described so far (Table. 2). Together with the increase in size of the ON, the HN is also greatly enlarged, compared to other reptantian Decapoda such as *Petrolisthes lamarckii *(Anomura, Porcellanidae), *Callianassa australiensis *(Thallasinidea, Callianassidae), *Carcinus maenas *Linnaeus, 1758 (Brachyura, Portunidae), *Gecarcoidea natalis *(Brachyura, Gecarcinidae), *Pagurus bernhardus *Linnaeus, 1758 (Anomura, Paguridae) and *Coenobita clypeatus *(Anomura, Coenobitidae); [40,43,47] and Krieger (unpublished data). Although our cell counts have to be considered estimates, cell cluster (5) with 250,000 interneuronal somata and associated with the lateral protocerebrum, nevertheless must be considered the largest cell body cluster of the entire brain (Table. 1; 160.000 projection neurons in cluster (10)). These numbers compare well with estimated cell counts in the spiny lobster *Panulirus argus *[69]. Our morphometric data of the neuropil volumes also are rough estimates, but the size relation between the neuropils is a close approximation to reality. For a better comparison with other taxa, we used the same calculation method as in the seminal study by Beltz et al. [44]. Compared to its close relative, the terrestrial hermit crab *Coenobita clypeatus *[44], the olfactory neuropils in *B. latro *(374,000,000 μm^3^) are three times larger and the olfactory glomeruli occupy twice the volume (Table. 2). The olfactory glomeruli of *B. latro *are five to seven times longer than broad, and as noticed in *C. clypeatus *by Harzsch and Hansson [40], this elongation of glomeruli marks one extreme of glomerular architecture present in Decapoda, where glomeruli typically are not finger- but rather barrel-shaped (discussed in [40]). Such an elongated shape may be the best geometrical way to fit high numbers of glomeruli into the radial array typical of decapod olfactory neuropils.

Clearly, the olfactory neuropils and the gigantic hemiellipsoid neuropils are outstanding features of the *B. latro *brain. As mentioned above, the neuropils of the crustacean lateral protocerebrum have an integrating function for the multimodal mechanosensory, optic and olfactory inputs that they receive (reviews [42,80]). Along these lines, ablation experiments in the spiny lobster *Panulirus argus *suggest that the lateral protocerebrum is responsible for the discrimination between food sources and nonfood items as well as for the control of spatiotemporal aspects of feeding behavior [81-83]. What kind of olfactory input do these animals face in their natural environment and what do we know about their diet and feeding behavior? Adult *B. latro *are opportunistic omnivores who prefer the fruit and pith of fallen trees but who also will hunt for brachyuran land crabs which share their habitat [32]. There are many observations from a Christmas Island population (Indian Ocean) that the animals favor fruit and pith of the Lister's Palm *Arenga listeri *and that they form large feeding congregations around Lister's Palms when their fruits are ripe [37]. There has been much debate if the animals can in fact open undamaged coconuts but now there are several reports that *B. latro *does in fact make use of this attractive food item [37]. As mentioned above, electro-antennographic studies with the well developed first antennae demonstrated the capacity of this organ to detect volatile chemical information (review Hansson et al. [36]). Stensmyr et al. [37] concluded that their physiological experiments on *B. latro *point to a peripheral olfactory system as sensitive as the most sensitive general odor-detecting olfactory sensory neurons found in insects, and that therefore is well suited to explore the terrestrial olfactory landscape. In a set of behavioural experiments, these authors [37] have also analyzed the behaviour of a population of *B. latro *on Christmas Island toward baits with favoured food resources such as dead red crabs, coconut and pith of Lister's Palm but also with single odor compounds. The animals strongly reacted toward baits of the carcass odor dimethyl trisulfide and could detect the odor source from a distance of more than 50 m. Detection typically triggered an upwind search for the source, with the crabs constantly sampling the air by flicking their antennules. Furthermore, the animals effectively located the baits with food items whereas they ignored control baits [37]. Taken together, there is ample evidence that locating food items by odor is an important facet in the behavioural repertoire of these animals (review in [36]).

On Christmas Island *B. latro *undertake rhythmic reproductive migrations in that the females visit the coast to spawn (review [37]) similar to the endemic Christmas Island red crabs *Gecarcoidea natalis *(Brachyura; [84]). This means that females living on the central plateau of the island must travel up to 5 km to reach the coast. Recent observations (Hansson and Drew, unpublished observations) suggest that females undertake targeted migrations to coastal caves to copulate and that they remain in the caves while their extruded eggs mature before visiting the ocean to spawn (review [37]). Non- migratory movements are less well known. Recent studies using GPS loggers showed that during the study period (December 2008) ten out of the 12 animals tracked for more than five days ranged in an area less than 260 m^2^. Nevertheless, one male individual which was monitored over 32 days migrated from its home range to the coast (almost 2 km distance) and then used an almost identical trajectory back to its home range (Harzsch, Stensmyr, Erland, Hansson; unpublished data). Taken together these initial observations foster the realisation that the animals can orient very well on the island and somehow are able to remember the topography or certain landmarks to guide their migrations to the coast and back to their home range. The well documented ability of *B. latro *to frequently climb trees adds the third dimension to their locomotive repertoire. Terrestrial hermit crabs of the closely related genus *Coenobita *have a remarkable set of orientation mechanisms, including a celestial compass, visual landmarks, and wind direction to orient towards the coastline and to optimize their fleeing direction (e.g. [38,85]). Because *B. latro *has a comparably long life-span that is estimated to more than 40 years [86], the lateral protocerebrum with the gigantic hemiellipsoid bodies might not only be a multimodal integration centre but also a region of cognitive mapping as was suggested for other reptantian decapods [87]. Given the massive inputs from the olfactory lobes and from the visual neuropils to the hemiellipsoid bodies we may speculate about some link between odor source, visual landmarks and spatial cues that form a kind of integrative memory.

Any discussion about multimodal integration centers in the brains of arthropods would of course bring to mind the insect mushroom bodies as an obvious analogy to the robber crab hemiellipsoid bodies. It appears that the mushroom body lobes of the earliest insects mainly served mechano- and optosensory integration rather than olfaction [88,89]. The mushroom body calyx seems to have evolved later only after the ability to detect distant airborne odors was established, so that today the mushroom bodies of pterygote insects are thought to play an essential role not only in odor discrimination and formation of an olfactory memory [90] but also in the integration of visual, tactile and acoustic stimuli and coordinating behavioral repertoires [88,91]. Insect mushroom bodies are characterized by a massive input from projection neuron axons which have their dendrites in the glomeruli of the antennal lobes, a type of cell that, considering its connections, closely resembles the projection neurons of malacostracan crustaceans [71]. In insect mushroom bodies, the projection neuron axons contact the neurites of local interneurons, the Kenyon cells, in a rectilinear pattern. The functional equivalent to the Kenyon cells in the *B. latro *brain would be the 250,000 interneurons in cell cluster (5) of the lateral protocerebrum. Unfortunately, the cellular architecture of the hemiellipsoid bodies in Coenobitidae is not yet known in detail. Nevertheless, unpublished work in progress using a silver impregnation technique suggests a rectilinear arrangement of alternating neurite bundles in the terrestrial hermit *Coenobita clypeatus *(Strausfeld, Harzsch, Hansson; unpublished data) as is also apparent in our Bodian stained preparations. The hemiellipsoid bodies of Coenobitidae may therefore represent functional analogs to insect mushroom bodies.

Similar to *B. latro*, endemic Christmas Island red crabs *Gecarcoidea natalis *(Brachyura) do undertake pronounced reproductive migrations, and visual cues, polarized light and magneto-reception have been suggested as navigational mechanisms [84]. Radio tracking experiments revealed that some specimens traveled more than 4 km within 6 days to reach the shoreline and to spawn. The crabs seem to have specific shore destinations to which they return each year. Interestingly, when returning from the shore to the central plateau of the island, some red crabs appear to follow a route similar to the one they took during their downward migration [84], an observation that matches our initial GPS tracking data for *B. latro*. One major difference between these two species which share the same habitat is that the red crab olfactory system is poorly developed, both in terms of minute antennae and minute olfactory lobes when compared to similarly sized marine brachyuran crab species (Krieger, Hansson, Harzsch; unpublished data). Despite their impressive navigational skills, olfactory cues seem to play a minor role in the sensory world of red crabs. Our initial neuroanatomical data suggest that concurrent with their olfactory lobes their hemiellipsoid bodies are also comparatively small.

## Conclusions

In a previous study on the terrestrial hermit crab *Coenobita clypeatus *we have shown that the central olfactory pathway in these animals is well developed compared to other brain areas and matching behavioral observations suggest that these animals have evolved aerial olfaction [40]. The present study shows that *B. latro *has even more inflated the amount of neuronal substrate in the central olfactory pathway, and these morphological characteristics correlate well with the proven ability to detect volatile substances in these terrestrial anomurans (see [37-39]). The primary olfactory centers are the dominating neuropils of the medial brain in *B. latro*. The secondary olfactory centers (hemiellipsoid neuropils) are also large and organized into parallel neuropil lamellae. Thus, we suggest that the expansion of the HN may compensate for the reduction of the AcN as integrating neuropil which is obvious in both Anomura and Brachyura. The paired olfactory neuropils of *B. latro *are composed of more than 1,000 glomeruli, ca. 90,000 local interneurons and ca. 160,000 projection neurons per side. The secondary olfactory centers, the paired hemiellipsoid neuropils, are targeted by the axons of these olfactory projection neurons which make contact to ca. 250,000 interneurons associated with the hemiellipsoid neuropils with the result that the olfactory input could be analyzed by ca. 500,000 olfactory interneurons per side. For insects with well developed olfactory systems [71] the numbers of local interneurons amounts to between ca. 400 (*Manduca sexta*) and 4,000 (*Apis mellifera*) and that of projection neurons to ca. 800 - 900 (*M. sexta *and *A. mellifera*). Thus, the numbers of interneurons in the central olfactory pathway of *B. latro *surpasses that of insects by two orders of magnitude. In accordance with these interneuron numbers, the total neuropil volume of the honeybee brain (*A. mellifera*) with ca. 4.48 × 10^8 ^μm^3^ is more than six times smaller than in *B. latro *[92]. However, it is difficult to discuss such absolute volumes as long as a reference to absolute body size is not possible. In terms of percentage of total neuropil volume, the primary olfactory processing areas (antennal lobes, 2.17 × 10^7 ^μm^3^) in *A. mellifera *amount to ca. 4.84%. This is a comparatively low value in relation to the 40% of neuropil volume occupied by the central olfactory pathway in *B. latro*. Hanström [68] already noted that decapod crustaceans dedicate relatively more neuronal tissue in their brains to the analysis of chemical stimuli than is the case in insects when writing: (*"Einige der erzielten neuen Resultate der vorliegenden Arbeit können auch als festgestellte Tatsachen gelten. Solche sind: ... 2. der Nachweis der im Verhältnis zu den Crustaceen ungeheuer großen Entfaltung der Sehzentren der Insekten und der überraschend unbedeutenden Entwicklung der Riechzentren derselben" *Some of the new results presented here can be considered established facts. These are: ... 2. evidence for the in relation to crustaceans enormous enlargement of the visual centers of insects and their surprisingly unimportant development of the olfactory centers).

Such comparison has to consider that the entire life span of the giant robber crab is much longer (40 years and more) than that of bees and that their olfactory pathway may not only serve the purpose of sensory analysis but also the formation of olfactory memory. Furthermore, it recently has been suggested that brain size may be less related to a complex behavioral repertoire and cognitive capacity than has been generally assumed [93]. These authors propose that "While *some *increases in brain size will affect cognitive capacity, many increases in certain brain areas - especially those involved in sensory and motor processing - produce only quantitative improvements: more detail, finer resolution, higher sensitivity, greater precision - in other words, *more of the same*." Miniaturization as seen in many insects may evoke the adaptive strategy to tune a brain to operate more economically and efficiently and to perform its task with less redundancy of neuronal circuits [93,94]. The brain of *B. latro *may suffer far less from limited energy resources than insect brains do. Therefore it may be less economical in performing the same olfactory tasks and need more neuronal hardware to do so. However, let us consider the numbers of olfactory glomeruli in the primary olfactory centers which in insects are considered a good estimate of the number of olfactory receptor proteins (ORs) expressed in antennal olfactory sensory neurons and therefore provides insights into the odor space that certain species can analyze. For example, *Drosophila melanogaster *expresses 62 functional olfactory receptor proteins [95] and *Apis mellifera *170 [96]. Although in crustaceans it is unknown if a similar correlation exists - i.e. more olfactory glomeruli means a greater diversity of olfactory receptor proteins (if crustaceans have any of these proteins at all) - we would like to remind that with approximately 1,340, *B. latro*, together with members of the genus *Panulirus*, has the highest number of olfactory glomeruli among all malacostracan crustaceans studied so far. Does this suggest a high level of odor discrimination, a high level of sensitivity, or does it mirror an uneconomical olfactory system? Chittka and Niven [93] suggest that "Increases in the volume of central brain regions can occur through the replication of nearly identical circuits - most likely engaged in more parallel processing ..." and "Within large brains, additional parallel processing pathways and stages of serial processing allowing the computation of novel receptive fields may be added more easily that in insect brains where space may impose more severe constraints." So, in *B. latro*, do we face an example where a multiplication of parallel processing pathways in the olfactory lobe - hemiellipsoid body system may in fact serve "quantitative improvements: more detail, finer resolution, higher sensitivity, greater precision" [93]?

Whatever the answer to this question may be, we can conclude that aerial olfaction plays a major role in the behavioral repertoire of all members of the Coenobitidae. Future studies using backfill methods should reveal more details of the olfactory pathway in these animals. The organization of the optic neuropils and those neuropils associated with antenna 2 suggest that both *C. clypeatus *and *B. latro *have visual and mechanosensory skills that are comparable to those of other Decapoda. However, it would appear that contrary to many terrestrial members of the Brachyura [62] the Coenobitidae have not fine-tuned their visual senses, but instead sharpened their olfactory senses for orientation in their terrestrial habitat. This aspect makes this species an extremely attractive model to analyze the sense of smell in arthropods.

## Methods

### Nomenclature

The neuroanatomical nomenclature, used in this manuscript is based on Sandeman et al. [45] with some modifications adopted from Harzsch and Hansson [40]. We consistently use the terms hemiellipsoid-, olfactory- and accessory neuropil according to Sandeman et al. (45) but have to note that other publications since use the terms hemiellipsoid body, olfactory lobe and accessory lobe in a synonymous way. The traditional nomenclature of the optic neuropils lamina ganglionaris, medulla interna, medulla externa has been modified here as suggested by Harzsch [50] to lamina, medulla, and lobula.

### Silver impregnations

Specimens of *Birgus latro *used for silver impregnation staining were obtained from Christmas Island. The anterior of the cepaholothorax was removed from the animals and the exposed brains and fixed in cold, aged alcoholic Bouin's fixative for 2 to 3 days after which they were washed and dissected free from the surrounding tissues. They were dehydrated in an ethanol series, cleared in xylene and embedded in paraffin wax. 10 micron serial sections were mounted on glass slides and stained using a modification of the Holmes-Blest silver impregnation method [97] in which impregnation times were increased up to 24 hours and toned with a 2% gold solution.

### Immunochemistry

Brains of four adult *Birgus latro *were obtained from specimens that were freshly killed in the heavy road train traffic associated with phosphate mining on, Christmas Island, Indian Ocean in December 2008 with permission of the Australian wildlife protection authorities. The brains were dissected in phosphate buffered saline (0.1 M PBS, pH 7.4) then fixed in 4% PFA in 0.1 PBS, pH 7.4 at 4°C, stored in Eppendorf tubes and then transported to the Zoological Institute, University of Greifswald. For the immunohistochemical experiments, the brains were washed for 4 hours in several changes of PBS and then sectioned (100 μm) with a Carl Zeiss vibratome (Hyrax) at room temperature. The sections were then preincubated for 90 minutes in PBS-TX (1% Bovine-Serum-Albumine, 0.3% TritonX-100, 0.05% Na-acide, pH 7.4) followed by the overnight incubation of the primary antibodies at room temperature diluted in PBS-TX. We used the following antisera for the triple labeling experiment (three section series): polyclonal rabbit anti-allatostatin (in PBS-TX; final dilution 1:1000; Abcam; Cat. No. AB53956), and monoclonal mouse anti-synapsin "SYNORF1"antibody (in PBS-TX; final dilution 1:2000; DSHB). For labeling against SIFamide related peptides, the primary rabbit anti-SIFamide antibody (final dilution 1:12.000; [98]) was used on the fourth section series. Subsequently, all tissues were washed in several changes of PBS for 2 hours at room temperature and incubated in a mix of the secondary anti-rabbit Alexa Fluor 488 antibodies, secondary anti-mouse Cy3 antibodies and the nuclear dye bisbenzimide as a histochemical counterstain (0.05%, Hoechst H 33258) for another 4 hours. For SIFamide labelling, specimens were incubated in a secondary Alexa Fluor 488 goat anti-rabbit immunoglobulin (1:50, Invitrogen, Eugene, or, USA) overnight at 4°C. Finally, the tissues were washed for at least 2 hours in several changes of PBS at room temperature and mounted in Mowiol. The labelled tissues were viewed with a Nikon eclipse 90i microscope connected to a Nikon camera DS2-MBWc and analyzed with the computer system using NIS-Elements AR softwareDigital images were processed with the Lucia 4.82 software package (Laboratory Imaging Ltd.) and Photoshop Elements (Adobe). Pictures were optimized with the global picture enhancement features of Photoshop elements (brightness/contrast). The pictures, obtained from the SIFamide labeling were black-white inverted.

### Antibody specifity

#### Synapsin

The monoclonal mouse anti-*Drosophila *synapsin "SYNORF1" antibody (provided by E. Buchner, Universität Würzburg, Germany) was raised against a *Drosophila *GST-synapsin fusion protein and recognizes at least four synapsin isoforms (ca. 70, 74, 80, and 143 kDa) in western blots of *Drosophila *head homogenates [99]. In western blot analysis of crayfish homogenates, this antibody stains a single band at ca. 75 kDa (see [55]). Harzsch and Hansson, [40] conducted western blot analysis comparing brain tissue of *Drosophila *and *Coenobita*. The antibody provided identical results for both species staining one strong band around 80-90 kDa and a second weaker band slightly above 148 kDa (see [40]). Their analysis strongly suggests that the epitope which SYNORF 1 recognizes is strongly conserved between the fruit fly and the hermit crab. Similar to *Drosophila*, the antibody consistently labels brain structures in representatives of all major subgroups of the malacostracan crustaceans (see [40,44,100-103]) in a pattern that is consistent with the assumption that this antibody does in fact label synaptic neuropil in Crustacea. In the crustacean first optic neuropil (the lamina), synapsin labeling is weak compared to the other brain neuropils [40,103]. Similarly, in *Drosophila *labeling of the lamina is weak because photoreceptors R1-R6 which have their synapses in the lamina contain very little of the presently known synapsin homolog isoforms [99]. The antibody also labels neuromuscular synapses both in *Drosophila *and in Crustacea [103]. These close parallels in the labeling pattern of SYNORF1 between *Drosophila *and various Crustacea strengthen the claim that it also recognizes crustacean synapsin homologs. Beyond the arthropods, this antibody even labels synaptic neuropil in ancestral taxa of protostomes such as the Chaetognatha [104,105] and Plathelminthes [106] suggesting that the epitope that this antiserum recognizes is highly conserved over wide evolutionary distances.

#### SIFamid

Crustacean-SIFamide is a 1,381-Da peptide that has been identified in the crayfish *P. clarkii *by topological mass spectrometry analysis in combination with MALDI-TOF MS using slices of tissues, chromatographic purification from the extract of tissues, molecular cloning for the determination of the precursor structure, and capillary liquid chromatography tandem mass spectrometry (LC-MS/MS) analysis for elucidation of its posttranslational modifications ([98,107]. The cDNA of this peptide has been characterized to encode a 76 amino acid precursor protein that contains a signal sequence, one copy of GYRKPP FNGSIFG and one additional peptide. Initial reverse transcriptase polymerase chain reaction (RT-PCR) analysis had demonstrated the presence of mRNA of this neuropeptide throughout the crayfish brain. Furthermore, direct MALDI-TOF MS analysis with microdissectates showed crustacean-SIFamide to be present in the olfactory neuropils, accessory neuropils, olfactory neuropil cells, anterior medial cells and posterior medial cells. The antigen used for generation of the antiserum (injection into rabbit) was (Cys)GYRKPPFNGSIF-CONH2 conjugated to bovine serum albumin (BSA) by the material basis set (MBS) method with NH2 [98]. Initial immunolocalization experiments with this antiserum in the crayfish [52] revealed a labeling pattern that closely corresponded to the experimental results of the aforementioned mass spectrometric morphology for crustacean-SIFamide [98]. More specifically, mass spectrometry analysis demonstrated that positively immunostained structures such as the lateral cell cluster 10, which houses olfactory projection neurons [43,47], in fact included the crustacean-SIFamide molecule. In subsequent experiments, the specificity of the antiserum was further substantiated. A digoxigenin-labeled antisense RNA probe prepared by in vitro transcription with partial preprotein cDNA as a template (1-313 in AB036713) was used for an in situ hybridization analysis of the crayfish brain [108]. The crustacean-SIFamide mRNA expression pattern was identical with the data obtained by the previous immunohistochemical analysis [98]. The antiserum also labels the projection neurons in another crayfish species [52] and the brachyuran crab *Libinia emarginata *[109]. Therefore, we expect that in *B. latro *the antiserum also recognizes crustacean-SIFamide. However, because but we cannot exclude the possibility that the antibody also binds to related peptides we will refer to "SIFamide-like immunoreactivity" throughout the paper.

#### Allatostatin

Allatostatin peptides share the conserved C-terminal sequence -YXFGL-NH2 [110] and are present in the nervous system of all insects analyzed so far (reviewed in [111]). Allatostatins are often colocalized with other transmitters, for example with GABA and other peptides in antennal lobes of moths [112,113]. Co-release with GABA is likely related to the inhibitory function of AST. The antiserum against *Diploptera punctata *(Pacific beetle cockroach) allatostatin I (Dip-allatostatin I, APSGAQRLYGFGL-amide) was provided by H. Agricola (Friedrich-Schiller Universität Jena, Germany). Vitzthum et al. [114] have characterized the Dip-allatostatin I antibody. It recognizes all Dip-allatostatins. No cross-reactivity was found with corazonin, CCAP, FMRFamide, leucomyosuppression, locustatachykinin 11, perisulfakinin, and proctolin as tested by non-competitive ELISA. Vitzthum et al. [114] showed that preadsorption of the diluted antisera against Dip-allatostatin I, GMAP and *Manduca sexta *allatotropin with 10 μM of their respective antigens abolished all immunostaining in brain sections of *Schistocerca gregaria*. For Crustacea, the antiserum was shown to label neurons in the central complex of the crayfish *Cherax destructor *[53]. However, because but we cannot exclude the possibility that the antibody also binds to related peptides in *B. latro*, we will refer to "allatostatin-like immunoreactivity" throughout the paper.

### Morphometric Analysis

For morphometry, aesthetasc numbers, cell numbers of the cell clusters (9), (10) and (11) were determined and the volumes of these cell clusters, the olfactory neuropils, the accessory neuropils, the hemiellipsoid neuropils and the neuropil volume of the entire brain were calculated.

To determine the neuropil volumes from the immunostained section of one animal, each section was viewed with a Nikon eclipse 90i microscope connected to a Nikon camera DS2-MBWc and analyzed with the computer system using NIS-Elements AR software. All synapsin labeled areas were selected and measured for each neuropil, except the AcN, and transferred into a Microsoft Excel table. Finally, the sum of all areas for each neuropil was multiplied by the section thickness of 100 μm to provide a total volume of the neuropils. Assuming that the accessory neuropil has an idealized spherical shape, the volume of the AcN was calculated *via *its diameter.

To obtain a mean value of a single olfactory glomerulus, the lengths and the cross sectional areas of ten randomly selected glomeruli of each olfactory neuropil were recorded. Next, the average of the cylindrical and the conical volume was calculated, because the olfactory glomeruli of *B. latro *have a conical as well as cylindrical shape. Finally, by dividing the total glomerular volume obtained before by the mean volume of a single glomerulus, the total glomerular number for each olfactory neuropil could be estimated.

For the cell counts of the cell cluster (10), (9/11) and the deutocerebral organ of another specimen, the series of silver-stained cross sections with a thickness of 10 μm per slice was examined. In different slices and in randomly selected subareas within the specific clusters, the average diameters of at least 30 cells per cluster were calculated. Based on an idealized spherical shape of the cells, the mean cell volume of each cluster was computed. Mean values for the packing densities were calculated for the ratio of the area filled by somata to a randomly pre-set area, at least five times per cluster. The maximum cell count for each slice was calculated by the quotient of the previously calculated cell cluster volume and the obtained mean cell volume. But due to the fact that the cells are not homogeneously distributed and only the cores are visible, the packing density was implicated into the calculation to obtain finally the estimated cell count for each cluster.

### 3D-Reconstruction

The silver-stained cross sections of 89 sections (10 μm) were viewed under a Nikon eclipse 90i microscope with a digital camera Nikon DS2-MBWc camera by using a 4× dry objective with a numeric aperture of 0.13. Each section was photographed as a mosaic of 2 × 3 single pictures, composed with Nikon's Imaging Software NIS-Elements AR. The three dimensional brain reconstruction of *B. latro *is based on this series and was prepared by using Bitplane's AutoAligner and Imaris 3 D reconstruction software. An exchange ratio of 1.587 was calculated from the proportion of 1.587 μm: 1 pixel. Therefore, the voxel *z*-dimension was set to 1.587 × 10 μm. The *x y *resolution of each stack was 2251 × 2990 pixels over a total scan area of 3.77 mm × 4.74 mm.

## List of Abbreviations

**I-VI**: noncolumnar olfactory neuropils; **A_I_Nv**: antenna I nerve; **A_II_Nv**: nerve of Antenna II; **AcN**: accessory lobe/neuropil; **AMPN**: anterior medial protocerebral neuropil; **AnN**: antenna II neuropil; **CA**: cerebral artery; **Cap**: cap neuropil of the hemiellipsoid neuropil; **CB**: central body neuropil; **CN**: columnar neuropil; **CO1, CO2**: core neuropils 1 and 2 of the hemiellipsoid neuropil; **DCF**: deutocerebral commissural fibers; **DGN**: serotonergic dorsal giant neuron; **DO**: deutocerebral organ; **HN**: hemiellipsoid neuropil; **ICh**: inner optic chiasm; **IL1, IL2**: intermediate layers 1 and 2 of the hemiellipsoid neuropil; **iT**: interconnecting tract; **La**: Lamina (lamina ganglionaris); **LAN**: lateral antenna 1 neuropil; **Lo**: lobula (medulla interna); **LoP**: lobula plate; **lPC**: lateral protocerebrum; **MAN**: median antenna I neuropil; **Me**: Medulla (medulla externa); **mF**: median foramen; **mPC**: Medial protocerebrum; **OC**: oesophageal connectives; **OCh**: outer optic chiasm; **OGT**: olfactory globular tract; **OGTN**: olfactory globular tract neuropil; **OGTNa**: accessory olfactory globular tract neuropil; **ON**: olfactory lobe/neuropil; **ONv**: optic nerve; **OT**: optic tract; **PB**: protocerebral bridge; **pF**: posterior foramen; **PMPN**: posterior medial protocerebral neuropil; **PT**: protocerebral tract; **TM**: terminal medulla; **TN**: tegumentary neuropil; **TNv**: tegumentary nerve; **X**: chiasm of the olfactory globular tract.

## Competing interests

The authors declare that they have no competing interests.

## Authors' contributions

SH and BSH conducted the sampling, preparation and fixation of brains on Christmas Island for the immunohistochemical experiments. JK carried out most of the immunohistochemical experiments, performed the 3 D reconstruction, and the microscopic and morphometric analysis. SH carried out the SIFamide immunolocalization experiment including microscopic analysis. RES and DCS obtained, sectioned, and stained the brain for the Bodian series. JK drafted the main part of the manuscript and all other authors assisted in drafting the manuscript. All authors read and approved the final manuscript.
